# ASWBoost: Classification algorithm for noisy and imbalanced data based on parametric exponential loss

**DOI:** 10.1371/journal.pone.0356889

**Published:** 2026-08-27

**Authors:** Fei Meng, Mei Yan, Hang Liu, Wan Xu, Hao Li

**Affiliations:** 1 School of Mathematics, Yunnan Normal University, Kunming, China; 2 Yunnan Key Laboratory of Modern Analytical Mathematics and Applications, Yunnan Normal University, Kunming, China; 3 School of Big Data, Yunnan Agricultural University, Kunming, China; Tishk International University, IRAQ

## Abstract

AdaBoost, a classical boosting ensemble algorithm, is widely applied for its strong classification performance. However, its standard exponential loss is highly sensitive to outliers, prone to overfitting, and inherently biased toward the majority class under class-imbalanced settings, degrading overall performance. To address these limitations, ASWBoost, a robust boosting algorithm, is proposed by introducing a parameter θ to modify the exponential loss. New update rules for base classifier weights and training sample probability distributions are derived, enabling adaptive adjustment of sample weights. The training error upper bound of the proposed algorithm is theoretically proven, and the impact of θ on its convergence properties is rigorously analyzed. Extensive experiments on synthetic and OpenML datasets demonstrate robust performance across Balanced Accuracy, G-mean, Macro-F1, and AUC. Critically, statistical analyses confirm that ASWBoost significantly outperforms AdaBoost, CS-AdaBoost, GBDT, SMOTEBoost, and RUSBoost, with overall performance comparable to XGBoost.

## 1. Introduction

Within the framework of Boosting ensemble learning [1], AdaBoost [2] can boost weak classifiers into a strong classifier without prior knowledge. Its superior classification performance has attracted widespread attention in the machine learning community. This ensemble mechanism provides AdaBoost with robust generalization capabilities [3], demonstrating highly competitive classification performance across various critical domains, including text classification [4], feature selection [5], object recognition [6], spam filtering [7] and face recognition [8].

However, because AdaBoost relies on an exponential loss function, it is highly sensitive to datasets containing noise [9]. Therefore, enhancing its noise tolerance is of significant importance for addressing practical application problems. Specifically, the AdaBoost algorithm optimizes the expected risk primarily by minimizing the exponential loss function, L(y,f(x))=exp(−yf(x)) [10], and fitting an additive model in a forward stagewise manner within the function space [11]. When noise is present in the training data, AdaBoost assigns excessively high weights to hard-to-classify noisy samples, inevitably leading to model overfitting [12]. Dietterich et al. [13] compared three ensemble algorithms—AdaBoost, Random Forest, and Bagging—and their results indicated that while AdaBoost consistently outperforms the other two in noise-free environments, its performance degrades significantly and falls behind both Random Forest and Bagging when noisy samples are introduced into the data. To mitigate this issue, Friedman et al. [10] proposed a series of representative AdaBoost variants that utilize adaptive Newton steps to optimize different loss functions. This approach ensures a smoother decrease in the training error of the optimal base learner, preventing excessive weight increases for misclassified samples and thereby reducing the algorithm’s sensitivity to noise. Furthermore, Kalai et al. [14] investigated robust base classifiers and boosting algorithms, finding that although robust classifiers converge faster than AdaBoost in the presence of label noise, their overall robustness remains insufficient. Consequently, Freund et al. [15] introduced a non-convex loss function incorporating a time variable based on the concept of Brownian motion, defined as $L\left(p,t\right)=1-erf\left[\frac{\rho -\mu \left(t\right)}{\sigma \left(t\right)}\right]$. Although this enhanced the robust capabilities of the algorithm, the non-convexity sacrifices the desirable properties of convex optimization, resulting in unstable training and high sensitivity to hyperparameters. The SPLBoost algorithm proposed by Wang et al. [16] integrated a self-paced learning mechanism into the AdaBoost framework, effectively enhancing the algorithm’s robustness. Its loss function is formulated as $\underset{f.v}{min}\sum_{i=1}^{N}\nu_{i}L\left(y_{i},f\left(x_{i}\right)\right)-\lambda \sum_{i=1}^{N}\nu_{i}$. However, this method requires complex explicit regularization terms for sample selection, leading to high computational costs. Additionally, multiple subsequent studies [17] have continuously focused on improving the robustness of boosting algorithms in noisy environments. Cao et al. [18] proposed a noise-detection based AdaBoost (ND-AdaBoost) that utilizes a k-nearest neighbor pre-filtering mechanism to identify and penalize mislabeled data. While effective, it relies on external distance metrics and separate preprocessing steps rather than an inherently robust loss function. More recently, Xing et al. [19] introduced a bounded exponential loss function to improve the AdaBoost ensemble of One-Class Support Vector Machines (OCSVMs). Although this bounded loss significantly reduces the negative impact of outliers, it is specifically tailored for one-class novelty detection and alters the convexity properties, complicating its direct application to standard multi-class imbalanced classification tasks.

AdaBoost also exhibits significant limitations when processing class-imbalanced datasets [20–22]. The primary reason for this substantial degradation in classification performance is that, while iteratively adjusting sample weights to focus on misclassified samples, the algorithm fails to account for the uneven distribution of class labels. Consequently, the algorithm struggles to effectively learn the features of the minority class, ultimately resulting in decision boundaries that are biased toward the majority class. To address this, Wang et al. [23] employed a synergistic mechanism of feature selection and weighted non-negative matrix factorization, enhancing AdaBoost’s ability to handle misclassified samples. Xiao et al. [24] integrated clustering algorithms to partition the dataset into distinct clusters, training weak classifiers separately before ensembling them, which effectively improved overall performance. The “AdaImC” algorithm proposed by Bei et al. [25] combined AdaBoost with cost-sensitive learning to enhance predictive accuracy. Kang et al. [26] fused AdaBoost with stacked DoG feature extraction, improving recognition accuracy and robustness in complex environments by extracting common features. While these studies primarily approach the issue from the perspectives of feature selection and data preprocessing, their generalization capabilities remain limited when the intrinsic distribution of the data changes. Among these approaches, cost-sensitive learning has proven effective for handling imbalanced classification or asymmetric misclassification costs. However, the loss function of Cost-Sensitive Boosting proposed by Fan et al. [27], defined as $L_{cs}=\sum_{i=1}^{N}C_{i}exp\left(-y_{i}f\left(x_{i}\right)\right)$, relies on a static cost matrix, making it susceptible to overfitting on minority class outliers. The AWABoost algorithm proposed by Wang et al. [28] applies a heuristic adjustment solely during the weight update step using $\varphi \left(\theta \right)=\theta +(1-\theta )\left(1-y_{i}G_{m}\left(x_{i}\right)\right)$. By inserting a parameter control function into the weight update process, it limits excessive weight growth from a procedural standpoint. This method relies heavily on empirical procedural constraints and lacks rigorous theoretical derivation based on objective function consistency, resulting in insufficient theoretical completeness.

To overcome the limitations, this paper proposes a robust boosting algorithm named ASWBoost. The algorithm strictly adheres to the framework of forward stagewise additive modeling, discarding heuristic fixed cost matrices and complex explicit regularization terms. By introducing a parameter θ to improve the traditional exponential loss function, formulated as $L\left(y,f\left(x\right),\theta \right)=exp\left[-yf\left(x\right)\left(1+\theta \left(\frac{1-y_{i}G_{m}\left(x_{i}\right)}{\text{2}}\right)\right)\right]$, it ensures the theoretical completeness and robustness of the algorithm in both noisy and imbalanced environments. Furthermore, it theoretically preserves the desirable properties of convex optimization. Through the parameter θ, the algorithm achieves adaptive adjustment of sample weights, thereby striking an effective balance between enhancing the feature learning of minority classes and suppressing the interference of noisy samples.

The main contributions of this paper are as follows:

1. **Improvement of the Exponential Loss**: The traditional exponential loss function is modified by introducing a parameter θ.
2. **Derivation of Updating Mechanisms**: Novel updating formulas for both the weights of the base classifiers and the probability distribution of the training samples are derived, enabling parameterized adjustment of sample weights.
3. **Proof of the Error Upper Bound**:The upper bound on the training error for the proposed algorithm is rigorously proven, guaranteeing its convergence.
4. **Empirical Validation**: Extensive experiments on a synthetic dataset and various OpenML medical datasets demonstrate that ASWBoost outperforms standard AdaBoost and other variant algorithms in terms of Balanced Accuracy, G-mean, Macro-F1, and AUC.

## 2. Preliminaries

### 2.1. The AdaBoost Algorithm

The core mechanism of the AdaBoost algorithm lies in training a series of weak classifiers by continuously adjusting sample weights. Specifically, in each iteration, it increases the weights of misclassified samples and decreases the weights of correctly classified ones. This strategy forces subsequent classifiers to focus more on hard-to-learn samples, thereby progressively improving the classification performance of the overall ensemble model.

**Algorithm 1.** AdaBoost

***Input***: A set of training samples with labels $\left\{\left(x_{\text{1}},y_{\text{1}}\right),\cdots ,\left(x_{N},y_{N}\right)\right\}$, a weak learning algorithm, and the maximum number of iterations *M*.

***Initialize***: The weights of the training samples: $D_{\text{1}}=w_{i}^{\text{1}}\leftarrow (1/N,\cdots ,1/N)$.

***For*** m = 1 **to** *M*:

(a) Learn a base classifier using the training set with weight distribution $D_{m}:$

    $G_{m}\left(x\right):\chi \to \left\{-1,+1\right\}$.

(b) Calculate the classification error rate of $G_{m}\left(x\right)$: $e_{m}\leftarrow \sum_{i=1}^{N}w_{mi}I\left(G_{m}\left(x_{i}\right)\neq y_{i}\right)$.

(c) Calculate the coefficient of $G_{m}\left(x\right)$: $\alpha_{m}\leftarrow \frac{\text{1}}{\text{2}}ln\frac{1-e_{m}}{e_{m}}$.

(d) Update the weight distribution $D_{m+1}$: $w_{m+1,i}\leftarrow \frac{w_{mi}}{Z_{m}}exp\left(-\alpha_{m}y_{i}G_{m}\left(x_{i}\right)\right),i=$

1,⋯,N. Where, $Z_{m}$ is a normalization constant, and $\sum_{i=1}^{N}w_{m+1,i}=1$.

***Output***: Final classifier $G\left(x\right)\leftarrow sign\left(\sum_{m=1}^{M}\alpha_{m}G_{m}\left(x\right)\right)$

### 2.2. Standard exponential loss and margin

From a statistical perspective, the AdaBoost algorithm can be interpreted as a forward stagewise additive modeling process that minimizes an underlying exponential loss function [10]. For a binary classification task, the performance of the ensemble classifier $f\left(x\right)=\sum_{m=1}^{M}\alpha_{m}G_{m}\left(x\right)$ on a specific training instance $\left(x_{i},y_{i}\right)$ is quantitatively evaluated by the classification margin, defined as $y_{i}f\left(x_{i}\right)$. The sign of the margin indicates the correctness of the classification, while its absolute value $\left|y_{i}f\left(x_{i}\right)\right|$ represents the confidence level of the prediction [12]. The ultimate objective of standard AdaBoost is to minimize the empirical exponential loss over the entire training dataset:


$$\begin{array}{c} L_{exp}=\sum_{i=1}^{N}exp\left(-y_{i}f\left(x_{i}\right)\right) \end{array}$$ (1)

At the m-th iteration, minimizing this objective intrinsically translates into the weight updating rule $\omega_{m+1,i}\propto \omega_{m,i}exp\left(-y_{i}G_{m}\left(x_{i}\right)\alpha_{m}\right)$. While the strict convexity of the exponential loss guarantees fast convergence and a global optimum in clean datasets, it becomes a critical vulnerability in practical environments contaminated with noise. As delineated by the mathematical property of the exponential function, $L_{exp}$ assigns an unbounded, exponentially growing penalty to instances with large negative margins $\left(y_{i}f\left(x_{i}\right)\ll 0\right)$. In real-world datasets, these hard-to-classify instances are typically outliers or mislabeled noise. Consequently, the standard exponential loss forces the subsequent base learners to disproportionately focus on these anomalous samples, causing the decision boundary to severely overfit the noise and thereby degrading the generalization performance. This intrinsic mathematical limitation underscores the theoretical imperative for reshaping the exponential loss function, which constitutes the core motivation behind the proposed ASWBoost algorithm.

## 3. Theoretical Analysis of ASWBoost

### 3.1. Parameterized exponential loss function

The ASWBoost algorithm can be formalized as a specific instance of forward stagewise additive modeling. Specifically, the final model is an additive model composed of base classifiers, and it is optimized using a modified version of the traditional exponential loss function. ASWBoost employs an additive ensemble approach, constructing the final model through the linear superposition of multiple base classifiers.

***Theorem 1****:* The ASWBoost algorithm is modeled using the forward stagewise additive algorithm. It is derived by iteratively training each weak classifier, which serves as a basis function in the linear combination, through the minimization of a modified exponential loss function.

***Proof****:* In terms of constructing an additive model, the ASWBoost algorithm acts as a concrete implementation of the forward stagewise algorithm. The final ensemble strong classifier is exactly the additive model obtained when selecting weak classifiers as basis functions within the forward stagewise framework. Furthermore, the step-by-step process of training weak classifiers in ASWBoost corresponds perfectly to the sequential learning of basis functions in the forward stagewise algorithm. The loss function of the ASWBoost algorithm is formulated as follows:


$$\begin{array}{c} L\left(y,f\left(x\right),\theta \right)=exp\left[-yf\left(x\right)\left(1+\theta \left(\frac{1-y_{i}G_{m}\left(x_{i}\right)}{\text{2}}\right)\right)\right],\theta \in (-1,1) \end{array}$$ (2)

### 3.2. Update formulae for classifier and sample weights

Assume that after m-1 iterations, the forward stagewise algorithm has obtained $f_{m-1}\left(x\right)$:


$$\begin{array}{c} f_{m-1}\left(x\right)=\alpha_{\text{1}}G_{\text{1}}\left(x\right)+\cdots +\alpha_{m-1}G_{m-1}\left(x\right) \end{array}$$ (3)

In the m-th iteration, the algorithm computes $\alpha_{m},G_{m}\left(x\right)$ to update $f_{m}\left(x\right)$ as:


$$\begin{array}{c} f_{m}\left(x\right)=f_{m-1}\left(x\right)+\alpha_{m}G_{m}\left(x\right) \end{array}$$ (4)

The objective is to find $\alpha_{m}$ and $G_{m}\left(x\right)$ that minimize the loss of $f_{m}\left(x\right)$ on the training dataset:


$$\begin{array}{c} \left(\alpha_{m},G_{m}\left(x\right)\right)=arg\underset{\alpha ,G}{min}\sum_{i=1}^{N}\omega_{mi}\left[exp\left(-y_{i}\alpha G\left(x_{i}\right)\right)\left(1+\theta \left(\frac{1-y_{i}G\left(x_{i}\right)}{\text{2}}\right)\right)\right] \end{array}$$ (5)

Since $w_{mi}$ depends neither on α nor on G, it acts as a constant within the current minimization step. However, $w_{mi}$ depends on $f_{m-1}\left(x\right)$ and changes with each iteration. Minimizing Equation (5) yields the optimal base classifier $G_{m}^{*}\left(x\right)$ and its coefficient $\alpha_{m}^{*}$ for the ASWBoost algorithm. The detailed derivation consists of two main steps:

First, we solve for $G_{m}^{*}\left(x\right)$. For any α>0, the optimal $G_{m}\left(x\right)$ that minimizes the objective is:


$$\begin{array}{c} G_{m}^{*}\left(x\right)=\underset{G}{argmin}\sum_{i=1}^{N}\omega_{mi}I\left(y_{i}\neq G\left(x_{i}\right)\right) \end{array}$$ (6)

Where, $\omega_{mi}=exp\left[-y_{i}f_{m-1}\left(x_{i}\right)\left(1+\theta \left(\frac{1-y_{i}G_{m}\left(x_{i}\right)}{\text{2}}\right)\right)\right]$.The classifier $G_{m}^{*}\left(x\right)$ is the base classifier $G_{m}\left(x\right)$ in the ASWBoost algorithm, as it is the base classifier that minimizes the weighted classification error rate on the training data in the m-th round.

Second, we solve for $\alpha_{m}^{*}$. The objective function is:


$$\begin{array}{c} T\left(\theta ,\alpha \right)=\underset{\alpha }{argmin}\sum_{i=1}^{N}\omega_{mi}exp\left[-y_{i}\alpha_{m}G_{m}\left(x_{i}\right)1+\theta \left(\frac{1-y_{i}G_{m}\left(x_{i}\right)}{\text{2}}\right)\right] \end{array}$$ (7)

Based on Equation (7), we solve for the optimal $\alpha_{m}^{*}$ as follows:


$$\begin{array}{c} \begin{array}{c} \underset{\alpha_{m}}{argmin}\sum_{i=1}^{N}\omega_{mi}e^{-y_{i}G_{m}\left(x_{i}\right)\alpha_{m}\left(1+\theta \left(\frac{1-y_{i}G_{m}\left(x_{i}\right)}{\text{2}}\right)\right)}\\ =\sum_{y_{i}=G_{m}\left(x_{i}\right)}\omega_{mi}e^{-\alpha_{m}}+\sum_{y_{i}\neq G_{m}\left(x_{i}\right)}\omega_{mi}e^{\alpha_{m}(1+\theta )}\\ =e^{-\alpha_{m}}\left(\sum_{i=1}^{N}\omega_{mi}-\sum_{y_{i}\neq G_{m}\left(x_{i}\right)}\omega_{mi}\right)+\sum_{y_{i}\neq G_{m}\left(x_{i}\right)}\omega_{mi}e^{\alpha_{m}(1+\theta )}\\ =e^{-\alpha_{m}}\sum_{i=1}^{N}\omega_{mi}-e^{-\alpha_{m}}\sum_{y_{i}\neq G_{m}\left(x_{i}\right)}\omega_{mi}+\sum_{y_{i}\neq G_{m}\left(x_{i}\right)}\omega_{mi}e^{\alpha_{m}(1+\theta )}\\ =e^{-\alpha_{m}}\sum_{i=1}^{N}\omega_{mi}+\left(e^{-\alpha_{m}(1+\theta )}-e^{-\alpha_{m}}\right)\sum_{y_{i}\neq G_{m}\left(x_{i}\right)}\omega_{mi.} \end{array} \end{array}$$

Applying *Fermat’s* theorem, we take the derivative of the objective function with respect to $\alpha_{m}$ and set it to zero:


$$\begin{array}{c} \frac{d}{d\alpha_{m}}=-e^{\alpha_{m}}\sum_{i=1}^{N}\omega_{mi}+\left[(1+\theta )e^{\alpha_{m}(1+\theta )}+e^{-\alpha_{m}}\right]\sum_{y_{i}\neq G_{m}\left(x_{i}\right)}\omega_{mi} \end{array}$$ (8)

Simplifying this yields the optimal $\alpha_{m}^{*}$:


$$\begin{array}{c} \alpha_{m}^{*}=\frac{\text{1}}{2+\theta }log\frac{1-e_{m}}{(1+\theta )e_{m}} \end{array}$$ (9)

Where $e_{m}$ is the weighted classification error rate:


$$\begin{array}{c} e_{m}=\frac{\sum_{i=1}^{N}\omega_{mi}I\left(y_{i}\neq G_{m}\left(x_{i}\right)\right)}{\sum_{i=1}^{N}\omega_{mi}} \end{array}$$ (10)

Thus, we have derived the optimal $\alpha_{m}^{*}$, which is identical to the $\alpha_{m}$ defined in the ASWBoost algorithm. From Equation (4) and $\omega_{mi}=e^{-y_{i}f_{m-1}\left(x_{i}\right)\left[1+\theta \left(\frac{1-y_{i}G_{m}\left(x_{i}\right)}{\text{2}}\right)\right]}$, we can further derive the sample weight update rule:


$$\begin{array}{c} \omega_{m+1,i}=\omega_{mi}e^{-y_{i}G_{m}\left(x_{i}\right)\alpha_{m}\left[1+\theta \left(\frac{1-y_{i}G_{m}\left(x_{i}\right)}{\text{2}}\right)\right]} \end{array}$$ (11)

Given the normalization factor is: $Z_{m}=\sum_{i=1}^{N}\omega_{mi}exp\left[-y_{i}G_{m}\left(x_{i}\right)\alpha_{m}\left(1+\theta \left(\frac{1-y_{i}G_{m}\left(x_{i}\right)}{\text{2}}\right)\right)\right]$

We obtain the normalized weight distribution:


$$\begin{array}{c} \omega_{m+1,i}=\frac{\omega_{mi}}{Z_{m}}e^{-y_{i}G_{m}\left(x_{i}\right)\alpha_{m}\left[1+\theta \left(\frac{1-y_{i}G_{m}\left(x_{i}\right)}{\text{2}}\right)\right]} \end{array}$$ (12)

The complete training procedure of the ASWBoost algorithm is outlined in Algorithm 2.

**Algorithm 2.** ASWBoost

***Input***: A set of training samples with labels $D=\left\{\left(x_{\text{1}},y_{\text{1}}\right),\cdots ,\left(x_{N},y_{N}\right)\right\}$, a weak learning algorithm, and the maximum number of iterations *M*, θ<0: noise handling, θ>0: imbalance handling.

***Initialize***: the weight of the training samples: $D_{\text{1}}=w_{i}^{\text{1}}\leftarrow 1/N,i=1,\cdots N.$

***For*** m = 1 **to** *M*:

(a) Learn a base classifier using the training set with weight distribution $D_{m}:G_{m}\left(x\right):\chi \to \left\{-1,+1\right\}$.

(b) Calculate the classification error rate of $G_{m}\left(x_{i}\right)$: $e_{m}\leftarrow \sum_{i=1}^{N}w_{mi}I\left(G_{m}\left(x_{i}\right)\neq y_{i}\right)$.

(c) Calculate the coefficient of $G_{m}\left(x\right)$: $\alpha_{m}\leftarrow \frac{\text{1}}{2+\theta }log\frac{1-e_{m}}{(1+\theta )e_{m}}$.

(d) Update the weight distribution     $D_{m+1}$:     $w_{m+1,i}\leftarrow \frac{\omega_{mi}}{Z_{m}}exp\left[-y_{i}G_{m}\left(x_{i}\right)\alpha_{m}\left(1+\theta \left(\frac{1-y_{i}G_{m}\left(x_{i}\right)}{\text{2}}\right)\right)\right],i=1,\cdots ,N.$

Where, $Z_{m}$ is a normalization constant, and $\sum_{i=1}^{N}w_{m+1,i}=1$.

***Output***: Final classifier $G\left(x\right)\leftarrow sign\left(\sum_{m=1}^{M}\alpha_{m}G_{m}\left(x\right)\right)$.

### 3.3. Training error upper bound analysis

***Theorem 2:*** (*Training Error Bound of ASWBoost*): The training error bound of the final classifier produced by the ASWBoost algorithm is given by:


$$\begin{array}{c} \frac{\text{1}}{N}\sum_{i=1}^{N}I\left(G\left(x_{i}\right)\neq y_{i}\right)\leq \frac{\text{1}}{N}\sum_{i}exp\left[-y_{i}f\left(x_{i}\right)\left(1+\theta \left(\frac{1-y_{i}G_{m}\left(x_{i}\right)}{\text{2}}\right)\right)\right]=\prod_{m=1}^{M}Z_{m} \end{array}$$ (13)

***Proof:*** When $G\left(x_{i}\right)\neq y_{i}$, it implies $y_{i}f\left(x_{i}\right)<0$, and consequently $exp\left(-y_{i}f\left(x_{i}\right)\right)\geq 1$. This directly establishes the first half of the inequality. Using the definitions from previous derivations, the second half regarding $Z_{m}$ can be deduced as follows:


$$\begin{array}{c} \begin{array}{c} \frac{\text{1}}{N}\sum_{i}\left[exp\left(-y_{i}f\left(x_{i}\right)\left(1+\theta \left(\frac{1-y_{i}G_{m}\left(x_{i}\right)}{\text{2}}\right)\right)\right)\right]\\ =\frac{\text{1}}{N}\sum_{i}\left[exp\left(-\sum_{m=1}^{M}y_{i}\alpha_{m}G_{m}\left(x_{i}\right)\left(1+\theta \left(\frac{1-y_{i}G_{m}\left(x_{i}\right)}{\text{2}}\right)\right)\right)\right]\\ =\sum_{i}Z_{\text{1}}\omega_{2,i}exp\left[-\sum_{m=2}^{M}y_{i}G_{m}\left(x_{i}\right)\alpha_{m}\left(1+\theta \left(\frac{1-y_{i}G_{m}\left(x_{i}\right)}{\text{2}}\right)\right)\right]\\ =Z_{\text{1}}\sum_{i}\omega_{2,i}\prod_{m=2}^{M}exp\left[-y_{i}G_{m}\left(x_{i}\right)\alpha_{m}\left(1+\theta \left(\frac{1-y_{i}G_{m}\left(x_{i}\right)}{\text{2}}\right)\right)\right]\\ =Z_{\text{1}}Z_{\text{2}}\cdots Z_{M-1}\sum_{i}\omega_{M,i}exp\left[-y_{i}G_{M}\left(x_{i}\right)\alpha_{M}\left(1+\theta \left(\frac{1-y_{i}G_{m}\left(x_{i}\right)}{\text{2}}\right)\right)\right]\\ =\prod_{m=1}^{M}Z_{m}. \end{array} \end{array}$$

This proves the upper bound of the training error for the modified exponential loss function. In each iteration, selecting a $G_{m}\left(x\right)$ with optimal classification performance ensures that $Z_{m}$ is minimized. Consequently, a smaller total training error bound is achieved, leading to a final classifier with minimized empirical error.

***Theorem 3:*** (*Impact of* θ *on the Training Error Bound*): Compared to the standard AdaBoost algorithm, when θ=0, ASWBoost is mathematically equivalent to AdaBoost. When 0<θ<1, the upper bound of the training error for the proposed algorithm is slightly larger, but it still decreases at an exponential rate. When −1<θ<0, the upper bound of the training error is smaller than that of AdaBoost, indicating superior convergence properties.

***Proof***: From the definition of $Z_{m}$, we have:


$$\begin{array}{c} Z_{m}=(1-e_{m})e^{-\alpha_{m}}+e^{\alpha_{m}(1+\theta )}e_{m}. \end{array}$$ (14)

Let $\gamma_{m}=\frac{\text{1}}{\text{2}}-e_{m}$.

a) When θ=0, the algorithm degenerates into the standard AdaBoost algorithm:

$$\begin{array}{c} \begin{array}{c} Z_{m}=(1-e_{m}){e_{m}}^{-\alpha_{m}}+{e_{m}}^{\alpha_{m}}\\ =2\sqrt{e_{m}(1-e_{m})}\\ =\sqrt{1-4{\gamma_{m}}^{\text{2}}}, \end{array} \end{array}$$

Using the Taylor expansion inequalities $e^{x}$ and $\sqrt{1-x}$ at x=0, we obtain $\sqrt{\left(1-4\gamma_{m}^{\text{2}}\right)}\leq exp(-2\gamma_{m}^{\text{2}})$. Consequently: $\prod_{m=1}^{M}Z_{m}=\prod_{m=1}^{M}\sqrt{1-4{\gamma_{m}}^{\text{2}}}\leq exp(-2\sum_{m=1}^{M}{\gamma_{m}}^{\text{2}})$. This demonstrates that the training error of the AdaBoost algorithm decreases exponentially.

b) When 0<θ<1, we have $Z_{m}=(1-e_{m})e^{-\alpha_{m}}+e^{\alpha_{m}(1+\theta )}e_{m}>(1-e_{m})e^{-\alpha_{m}}+e_{m}e^{\alpha_{m}}$. If we were to use the standard coefficient $\alpha_{m}=\frac{\text{1}}{\text{2}}log\frac{1-e_{m}}{e_{m}}$, the upper bound of ASWBoost would be strictly larger than standard AdaBoost. However, utilizing the optimal coefficient derived for the proposed algorithm, $\alpha^{*}\text{=}\frac{\text{1}}{2\text{+}\theta }ln\frac{1-e_{m}}{(1+\theta )e_{m}}$, the training error still decreases exponentially.

***Proof****:* When 0<θ<1, the true upper bound of the training error is:


$$\begin{array}{c} \begin{array}{c} \prod_{m=1}^{M}Z_{m}=\prod_{m=1}^{M}(1-e_{m})e^{-{\alpha_{m}}^{*}}+e_{m}e^{{\alpha_{m}}^{*}(1+\theta )}\\ \leq \prod_{m=1}^{M}(1-e_{m})e^{-\alpha_{m}}+e_{m}e^{\alpha_{m}(1+\theta )}\\ =\prod_{m=1}^{M}\sqrt{1-4{\gamma_{m}}^{\text{2}}}{\left(\frac{1+2\gamma_{m}}{1-\gamma_{m}}\right)}^{\frac{\theta }{\text{4}}}. \end{array} \end{array}$$

For the function $f\left(x\right)={\left(\frac{1+2x}{1-2x}\right)}^{\frac{\theta }{\text{4}}}$, its Taylor expansion at x=0 is:


$$\begin{array}{c} f\left(x\right)=1+\theta x+\frac{\text{1}}{\text{2}}\theta^{\text{2}}x^{\text{2}}+\left(\frac{4\theta }{\text{3}}+\frac{\theta^{\text{3}}}{\text{6}}\right)x^{\text{3}}+\left(\frac{4\theta^{\text{2}}}{\text{3}}+\frac{\theta^{\text{4}}}{24}\right)+O\left(x^{\text{5}}\right) \end{array}$$ (15)

The Taylor series of the exponential function $e^{\frac{\theta x}{1-2x}}$ serves as an upper bound for f(x), meaning $f\left(x\right)\leq e^{\frac{\theta x}{1-2x}}$. This leads to:


$$\begin{array}{c} \prod_{m=1}^{M}Z_{m}\leq exp\left(-2\theta \sum_{m=1}^{M}\frac{\gamma_{m}^{\text{3}}}{1-2\gamma_{m}}\right) \end{array}$$ (16)

This proves that even for 0<θ<1, the training error of ASWBoost continues to decrease at an exponential rate.

c) When −1<θ<0:

$$\begin{array}{c} \begin{array}{c} Z_{m}=(1-e_{m})e^{-\alpha_{m}}+e^{\alpha_{m}(1+\theta )}e_{m}\\ <(1-e_{m})e^{-\alpha_{m}}+e_{m}e^{\alpha_{m}}\\ =2\sqrt{e_{m}(1-e_{m})}\\ =\sqrt{1-4{\gamma_{m}}^{\text{2}}}. \end{array} \end{array}$$

When −1<θ<0 is negative, the upper bound of the training error for the proposed algorithm is strictly smaller than that of standard AdaBoost, resulting in superior convergence.

### 3.4. Explanation of the ASWBoost Algorithm

The sample weight updating mechanism in ASWBoost is critical, as it shapes the training data distribution for subsequent iterations. For a binary classification task, the updating rule can be explicitly expressed as:


$$\begin{array}{c} \omega_{m+1,i}=\left\{ \begin{array}{c} @l@\frac{\omega_{m,i}}{Z_{m}}exp(-\alpha_{m});\;G_{m}=y_{i}\\ \frac{\omega_{m,i}}{Z_{m}}exp\left(\alpha_{m}(1+\theta )\right);\;G_{m}\left(x_{i}\right)\neq y_{i} \end{array}\right. \end{array}$$ (17)

Building upon the foundation of AdaBoost, ASWBoost retains the core strategy of increasing the weight of misclassified samples and decreasing the weight of correctly classified ones. However, the magnitude of this weight adjustment is governed by the parameter θ∈(−1,1). This mechanism does not alter the underlying statistical distribution of the dataset; rather, it provides a new weight distribution in each iteration, guiding subsequent weak classifiers to focus on hard-to-classify samples.

**Fig 1:**
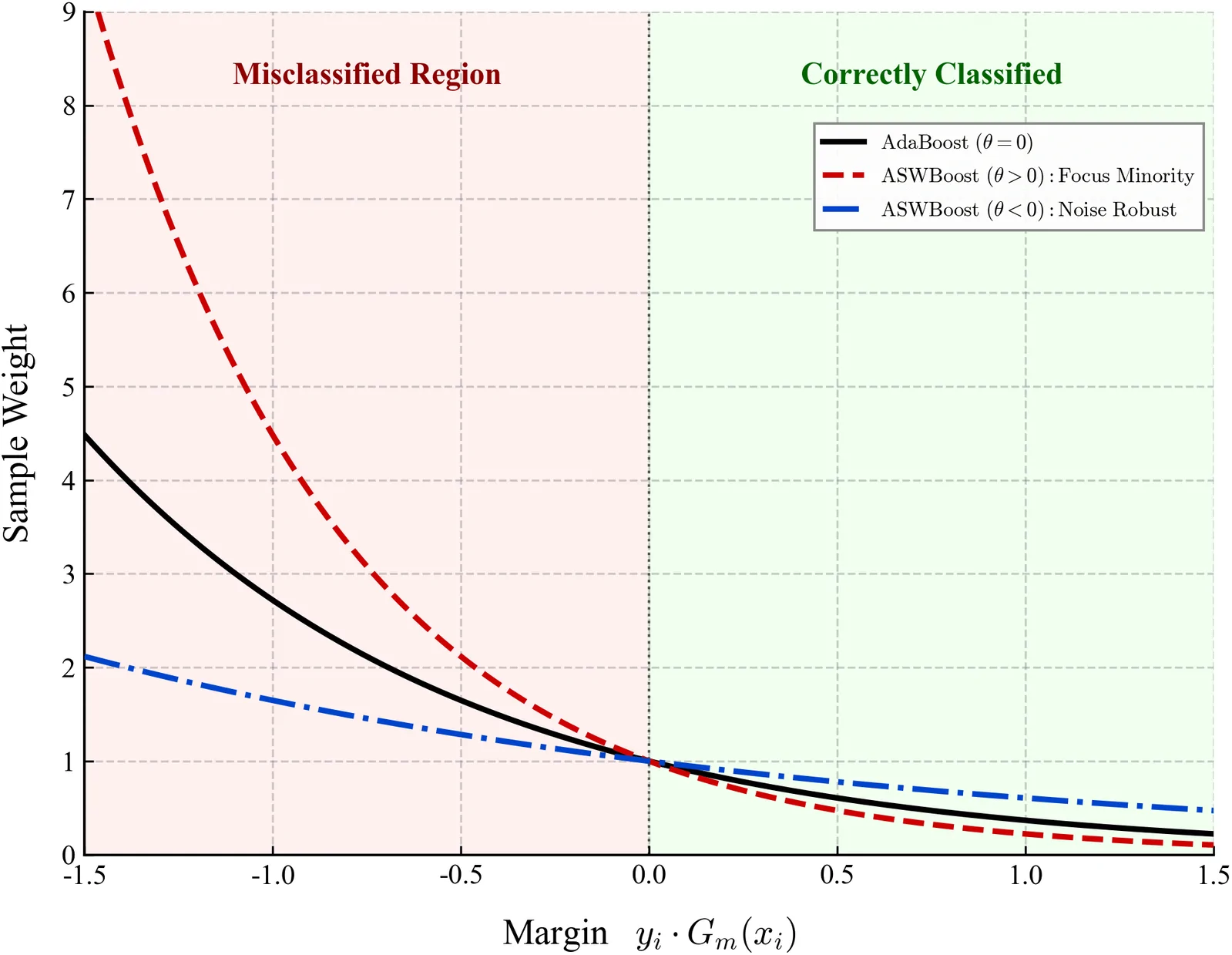
Impact of parameter θ on sample weight updates.

The parameter θ plays a pivotal role in this process. When θ=0, ASWBoost completely degenerates into standard AdaBoost. When θ>0, the magnitude of weight reduction for correctly classified samples remains consistent, but the penalty (weight increase) for misclassified samples is amplified. This explicitly directs the algorithm to focus more heavily on the minority class in extremely imbalanced datasets. Conversely, when θ<0, the weight increase for misclassified samples is attenuated, resulting in a more conservative updating scheme. This constraint effectively prevents the model from overfitting when dealing with noisy data, as the weights of hard-to-classify noisy samples are prevented from growing exponentially. The distinct weight updating mechanisms in noisy and imbalanced environments are illustrated in Fig 1.

### 3.5. Connection and distinction with existing robust boosting frameworks

To fully articulate the theoretical positioning of ASWBoost, it is essential to compare its mathematical mechanisms with recent advanced robust boosting frameworks, notably ND-AdaBoost [18] and BELF-AEOCSVMs [19]. All three algorithms fundamentally recognize that standard AdaBoost’s exponential loss function leads to overfitting by consistently assigning excessively high weights to hard-to-learn instances. However, their theoretical solutions differ significantly:

(1) Implicit vs. Explicit Noise Handling: ND-AdaBoost introduces an explicit noise-detection function $\phi_{t}\left(x_{n}\right)$ into the loss function, requiring external algorithms (such as k-NN or EM) to assign a noise label at each iteration. While this explicitly categorizes instances into four types, it incurs substantial computational overhead. In contrast, ASWBoost introduces the parameter θ to reshape the curvature of the loss function. This allows the algorithm to implicitly limit the weight penalty for large-margin misclassified instances without relying on any external distance metrics, thereby maintaining algorithmic efficiency.
(2) Convexity and Optimization: BELF-AEOCSVMs replaces the traditional loss with a bounded exponential loss function $\ell_{bexp}\left(u\right)=\xi \left[1-exp\left(-\eta \ell_{bexp}\left(u\right)\right)\right]$. While highly effective at neutralizing outliers, this bounded function is mathematically smooth but nonconvex, which complicates optimization and necessitates complex numerical methods like the Newton-Raphson approach. ASWBoost strictly preserves the desirable convexity of the exponential loss. By ensuring convexity, ASWBoost guarantees that the forward stagewise additive modeling (Theorem 1) converges stably to the global optimum, ensuring rigorous theoretical error bounds (Theorem 2) for standard decision trees.

Thus, ASWBoost acts as a theoretical bridge: it achieves the robust outlier-suppression capabilities seen in bounded methods and noise-detection methods, while retaining the mathematical simplicity and convexity of standard AdaBoost.

## 4. Experimental results and analysis

In this section, we evaluate the classification performance of the proposed ASWBoost algorithm on a synthetic dataset and 12 OpenML datasets. Furthermore, a *Friedman* test with post-hoc analysis is employed to determine whether statistically significant differences exist between ASWBoost and the baseline algorithms. To ensure the robustness of the experimental results, a stratified cross-validation framework is adopted. The outer loop utilizes a five-fold cross-validation, while the inner loop performs automatic optimization of the hyperparameter θ via nested grid search and three-fold cross-validation. All experiments are conducted using a fixed random seed of 42 to guarantee reproducibility.

### 4.1. Evaluation metrics

To comprehensively evaluate the model’s performance on imbalanced data and prevent biases dominated by the majority class, we utilize Balanced Accuracy, G-mean, Macro-F1, and Area Under the Curve (AUC) as evaluation metrics [29].

Balanced Accuracy is the arithmetic mean of the recall for each class:


$$\begin{array}{c} Balanced\;Accuracy=\frac{Sensitivity+Specificity}{\text{2}} \end{array}$$ (18)

G-mean comprehensively considers the model’s discriminative ability across all classes, achieving a high score only when the model performs well on all classes:


$$\begin{array}{c} G-mean=\sqrt{Sensitivity\times Specificity} \end{array}$$ (19)

Macro-F1 treats each class equally regardless of its frequency of occurrence, calculating the F1 score for each class and then averaging them:


$$\begin{array}{c} Macro-F1=\frac{\text{1}}{N}\sum_{i=1}^{N}F{\text{1}}_{i} \end{array}$$ (20)

AUC represents the probability that the model ranks a randomly chosen positive instance higher than a randomly chosen negative instance. Its value ranges from 0.5 to 1, with values closer to 1 indicating stronger discriminative ability.

### 4.2. Performance on synthetic datasets

The synthetic dataset is generated using the *make_classification* function in Python to simulate common data noise, feature redundancy, and class imbalance found in real-world scenarios. The dataset comprises 1,000 samples and 40 features, among which 25 are informative features, 10 are redundant features (random linear combinations of the informative features), and the remaining 5 are noise features drawn from a standard normal distribution *N* (0,1). The inter-class separability is controlled by setting *class_sep* = 1.5.

**Fig 2:**
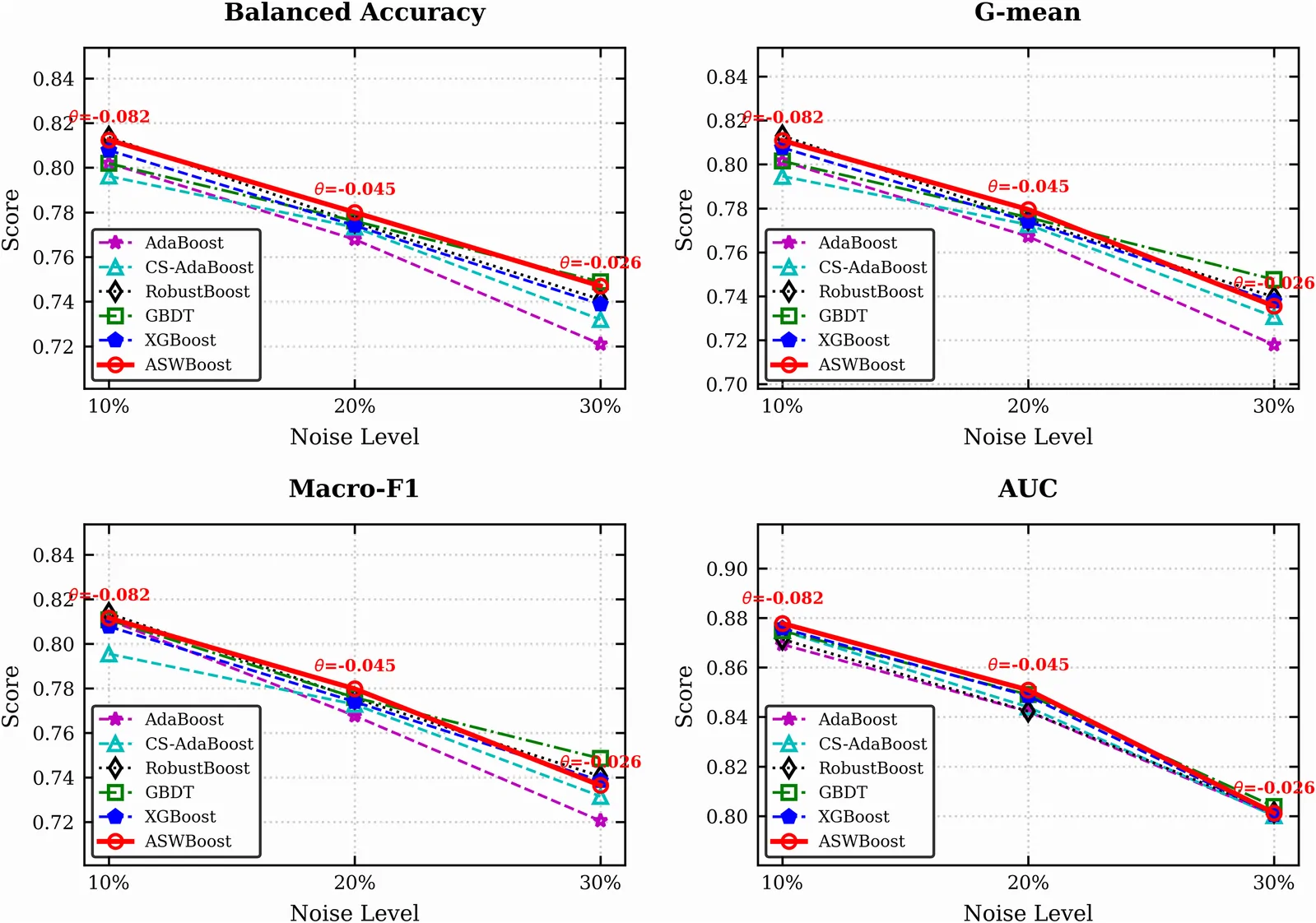
Performance comparison of different algorithms under varying noise levels on the synthetic dataset. (Figure Captions: Experimental performance of six algorithms across 10%, 20%, and 30% noise levels in terms of Balanced Accuracy, G-mean, Macro-F1, and AUC. The optimal values for θ are −0.082, −0.045, and −0.026, respectively).

The proposed ASWBoost algorithm is compared against standard AdaBoost, Cost-Sensitive AdaBoost (CS-AdaBoost), RobustBoost, GBDT, and XGBoost on this synthetic dataset. To evaluate the algorithm’s robustness in noisy environments, varying degrees of label noise are simulated by randomly flipping 10%, 20%, and 30% of the class labels. A grid search is employed to determine the optimal parameter θ and to investigate its relationship with the performance of ASWBoost. Fig 2 illustrates the comparative experimental results across different noise conditions.

As shown in the experimental results in Fig 2, the performance of AdaBoost and CS-AdaBoost degrades significantly in high-noise environments. This is primarily attributed to the weight allocation mechanism of the traditional exponential loss function for misclassified samples. During the iterative process, hard-to-classify noisy samples are assigned increasingly larger weights, leading the base learners to overfit the noise. In contrast, XGBoost and GBDT employ log-loss functions with second-order approximation optimization and incorporate regularization terms, making them more robust to higher ratios of label contamination. RobustBoost improves noise tolerance to some extent by designing a robust loss function; however, it still exhibits limitations in filtering high-dimensional noise features. ASWBoost preserves the convexity of the traditional exponential loss while mitigating the penalty intensity for small-margin samples by introducing the parameter θ. This effectively suppresses the abnormal expansion of weights for noisy samples during iterations. The experiments validate our theoretical premise: the optimal negative values of θ obtained through grid search alter the curvature of the exponential loss, restrict the excessive growth of weights for misclassified noisy samples, and thereby enhance the model’s robustness against outliers.

**Fig 3:**
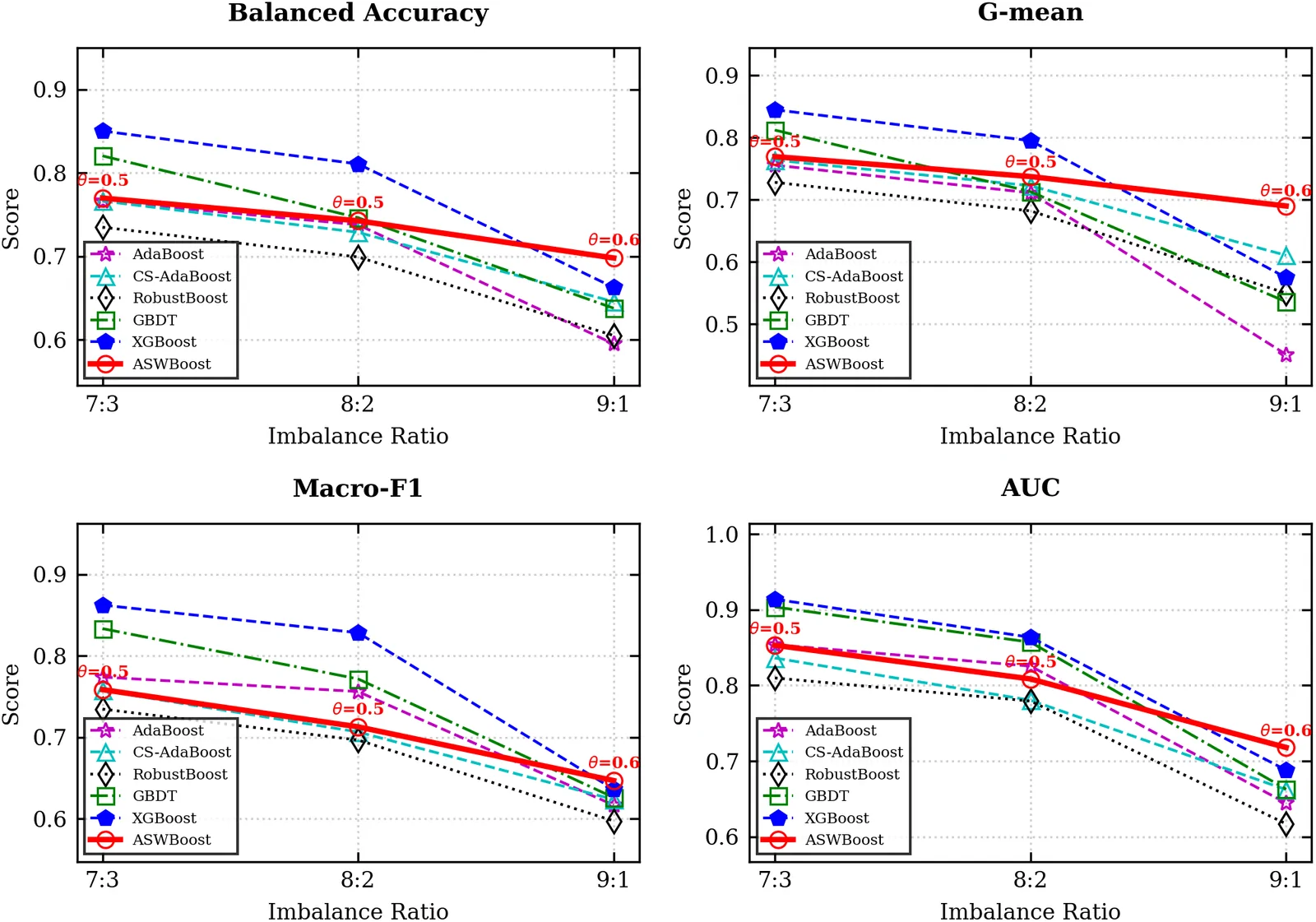
Generalization performance of various algorithms under different class-imbalance ratios on the synthetic dataset. (Figure Captions: Experimental performance of six algorithms across imbalance ratios of 7:3, 8:2, and 9:1, evaluated by Balanced Accuracy, G-mean, Macro-F1, and AUC. The optimal values of θ for these ratios are 0.5, 0.5, and 0.6, respectively).

To verify the generalization performance of ASWBoost on class-imbalanced data, three highly imbalanced datasets with negative class proportions of 90%, 80%, and 70% are constructed based on the synthetic data for experimental evaluation. The results are illustrated in Fig 3.

The experiments demonstrate that as the degree of imbalance intensifies, the performance of all models degrades to varying degrees. However, ASWBoost exhibits significant robustness in terms of G-mean and Balanced Accuracy. Particularly in highly imbalanced scenarios (8:2 and 9:1), it substantially outperforms standard AdaBoost and its variants, proving its superiority in balancing minority class recall and majority class precision. Although CS-AdaBoost increases attention to the minority class via a cost-sensitive factor, it fails to fundamentally mitigate the overfitting risks induced by exponential weight growth. GBDT and XGBoost show strong competitiveness under mild imbalance; however, under extreme imbalance, their global gradient optimization inevitably shifts toward the majority class, reflected by a noticeable decline in G-mean (which captures minority class recognition capability). When θ>0, ASWBoost no longer blindly pursues absolute correctness for every sample as standard AdaBoost does. Instead, through its margin-based weight updating mechanism, it emphasizes enhancing minority class recall, avoids misclassifying them as noise, and thereby strengthens the model’s generalization capacity.

### 4.3. Complexity analysis

Let N be the number of training samples, d be the feature dimension, M be the number of iterations, and the training complexity of the weak classifier be O(ψ(N,d)). The computational complexity of AdaBoost can be formalized as $O\left(VC\left(C)\cdot poly(R\left(C\right),1/\gamma^{\text{2}}\right)\right)$ [30], which primarily stems from conducting approximately $\tilde{O}(1/\gamma^{\text{2}})$ sequential iterations. Each iteration involves core steps such as training the weak classifier O(ψ(N,d)), calculating the weighted error rate O(N), and updating the sample weights O(N). Therefore, the total time complexity is O(M·(ψ(N,d)+N)). The proposed ASWBoost algorithm retains all the computational steps and data dependencies. The introduced parameter θ merely serves as a constant factor in the weight coefficient calculations and does not alter the asymptotic complexity order of the algorithm. Consequently, ASWBoost remains consistent with standard AdaBoost in both time and space complexity, with its space complexity also bounded by O(N+M).

To empirically validate the theoretical analysis, we conducted runtime experiments comparing ASWBoost with the baseline models on the synthetic dataset. The results are shown in Table 1.

**Table 1:** Computational time comparison of evaluated algorithms on the synthetic dataset.

Algorithm	Operating time (s)
AdaBoost	0.4911 ± 0.1332
CS-AdaBoost	0.1388 ± 0.0127
RobustBoost	0.2503 ± 0.0850
GBDT	0.5993 ± 0.1463
XGBoost	0.2813 ± 0.0210
ASWBoost	5.7516 ± 0.3282

Table 1 presents the average runtime and standard deviation of 5-fold cross-validation for each algorithm under identical experimental settings. The results indicate that the runtime of ASWBoost is significantly higher than that of the other algorithms. This is primarily attributed to the external hyperparameter tuning overhead. The experiment employed a nested cross-validation strategy with grid search to precisely optimize θ, thereby increasing the time cost of model training and evaluation. However, considering the enhanced robustness and generalization performance on noisy and imbalanced data, this increase in runtime is entirely justifiable and acceptable.

### 4.4. Performance on OpenML Datasets

To further validate the effectiveness of the ASWBoost algorithm in handling class-imbalanced data under real-world medical scenarios, comprehensive experiments were conducted on 12 datasets sourced from the OpenML repository (https://www.openml.org). As detailed in Table 2, these datasets encompass a diverse range of sample sizes, feature dimensions, and severe imbalance levels. For multi-class datasets (e.g., Hypothyroid, Cardiotocography, and Dermatology), the selected target class was treated as the positive class, while all remaining classes were aggregated into the negative class, effectively transforming them into highly imbalanced binary classification tasks.

**Table 2:** Characteristics and instance distributions of the 12 real-world OpenML datasets.

Data Set	Sample Number	Feature Number	Original Classes	Majority Instances (Negative)	Minority Instances (Positive)	Ratio (majority: minority)
Mammography	11183	6	2	10923	260	42.01:1
Diabetes	768	8	2	500	268	1.87:1
Breast Cancer	568	30	2	356	212	1.68:1
ILPD	583	10	2	416	167	2.49:1
Leukemia	72	7129	2	47	25	1.88:1
SPECT heart	267	22	2	212	55	3.85:1
Hypothyroid	3772	29	4	3541	231	15.33:1
Cardiotocography	2126	21	10	1655	471	3.51:1
Transfusion	748	4	2	570	178	3.20:1
Heart Failure	299	12	2	203	96	2.11:1
EEG Eye State	14980	14	2	13352	1628	8.2:1
Dermatology	366	34	6	305	61	5.0:1

During the data preprocessing stage, all features were standardized to ensure a mean of 0 and a standard deviation of 1. Regarding experimental parameter settings, the search space for the hyperparameter θ was strictly constrained between (−1, 1). The baseline models were implemented using the Scikit-learn library with default configurations, and the number of iterations was uniformly set to 50 for all boosting algorithms. To accurately reflect the characteristics and the exact imbalance severity of the data, Table 2 explicitly outlines the original number of classes, the specific number of instances in both the majority and minority classes, and the resulting imbalance ratio. All reported results in the subsequent analyses represent the averages derived from 5-fold cross-validation to ensure maximum reliability.

The real-world medical datasets detailed in Table 2 provide a rigorous testbed for evaluating algorithmic robustness under complex distributions. Specifically, the nature of the class imbalance originates from the epidemiological rarity of positive disease instances (e.g., *Mammography, Hypothyroid*) relative to healthy baselines, which inherently biases standard classifiers toward the majority class. Concurrently, the nature of the noise is dual-faceted: it comprises feature noise from instrumental artifacts (e.g., *EEG* sensor inaccuracies) and label noise driven by subjective diagnostic discrepancies. Although Macro-F1 was evaluated on synthetic data, it was omitted in real-world experiments because its inherent reliance on precision renders it excessively volatile and potentially misleading under extreme class skews. Consequently, we exclusively prioritized AUC, Balanced Accuracy, and G-mean to yield a more robust, prevalence-independent assessment of the model’s true discriminative capability in severely imbalanced medical scenarios.

**Fig 4:**
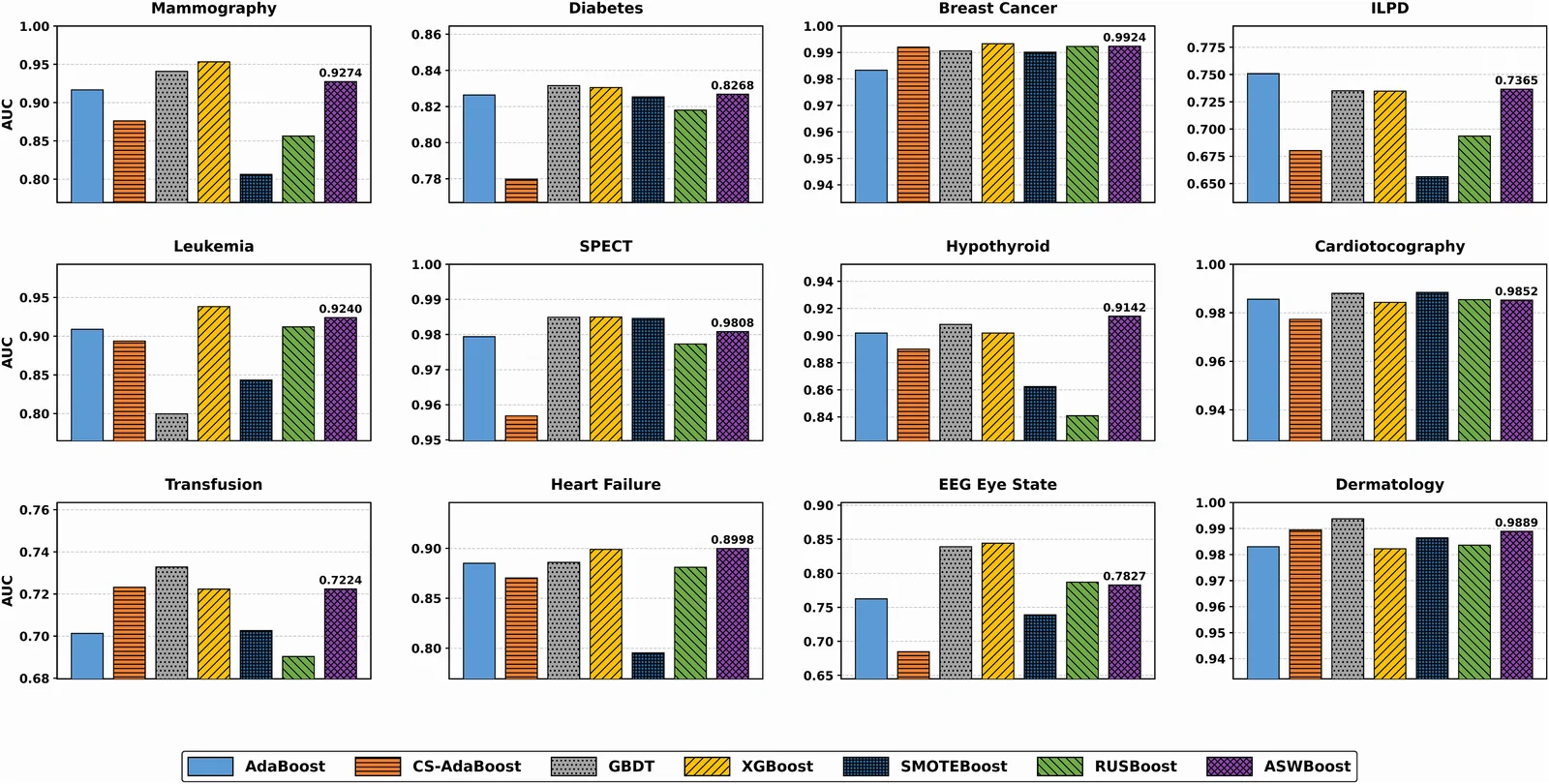
AUC evaluation of various algorithms across 12 real-world OpenML datasets.

Fig 4 presents the AUC comparisons across the 12 OpenML datasets. Overall, ASWBoost achieved optimal or near-optimal performance on the vast majority of the datasets. This indicates that ASWBoost, through its adaptive sample weighting mechanism, effectively overcomes the inherent flaws of standard AdaBoost, namely its sensitivity to noise and insufficient focus on minority classes.

Specifically, on highly imbalanced datasets such as *Mammography* and *Hypothyroid*, standard AdaBoost’s performance was restricted due to majority class dominance, while RUSBoost suffered fluctuations due to information loss from under-sampling. In contrast, ASWBoost effectively mitigated the interference from majority class samples via its adaptive weight adjustment mechanism. Its AUC values were significantly higher than those of CS-AdaBoost and SMOTEBoost, demonstrating its robustness under extreme distributions. Compared to GBDT and XGBoost, ASWBoost exhibited stronger competitiveness on datasets like *Pima Indians Diabetes*, *Heart Failure*, and *Indian Liver Patient*. This demonstrates that ASWBoost not only addresses the imbalance issue but also broadens the model’s generalization boundaries through a weighting strategy based on sample difficulty and distribution, effectively preventing overfitting.

**Fig 5:**
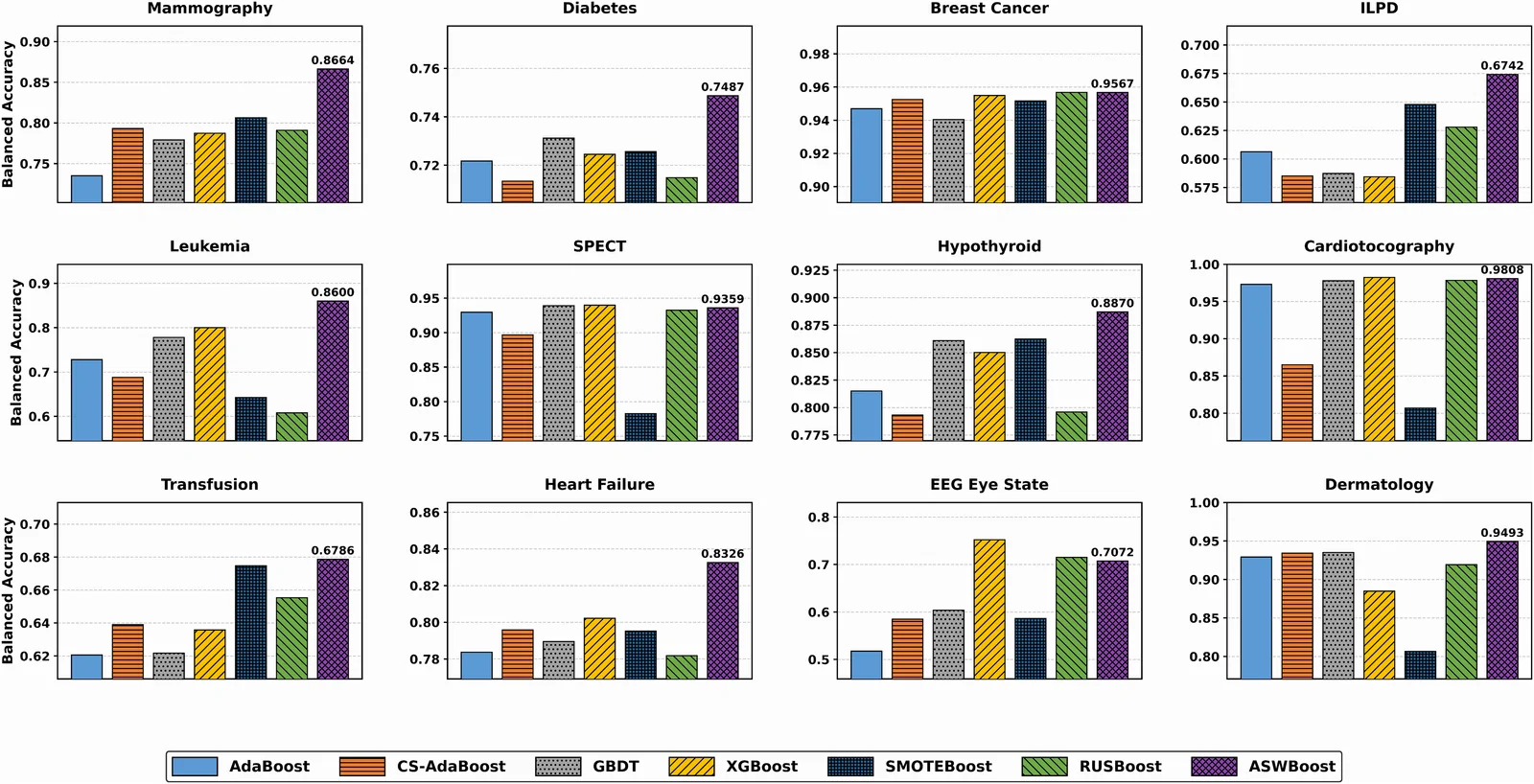
Balanced Accuracy evaluation of various algorithms across 12 real-world OpenML datasets.

Fig 5 further evaluates the decision bias and overall recall capability of the models on imbalanced datasets. The experimental results show that ASWBoost achieved the highest Balanced Accuracy on the majority of the datasets, validating its significant effectiveness in alleviating class imbalance bias.

ASWBoost secured the highest Balanced Accuracy on most datasets, including *Mammography*, *Indian Liver Patient*, *Leukemia*, and *Heart Failure*. Compared to the oversampling SMOTEBoost and the under-sampling RUSBoost, ASWBoost performed much better and more stably on datasets like *Hypothyroid* and *Blood Transfusion*. This suggests that the adaptive weighting mechanism of ASWBoost can preserve original information more effectively than simply altering the data distribution, thereby enhancing the discriminative power of the classification hyperplane. When compared against strong ensemble learners like XGBoost and GBDT, ASWBoost also demonstrated exceptional competitiveness. This further confirms that the algorithm successfully balances the classification accuracy of both minority and majority classes by optimizing the sample weight distribution.

**Fig 6:**
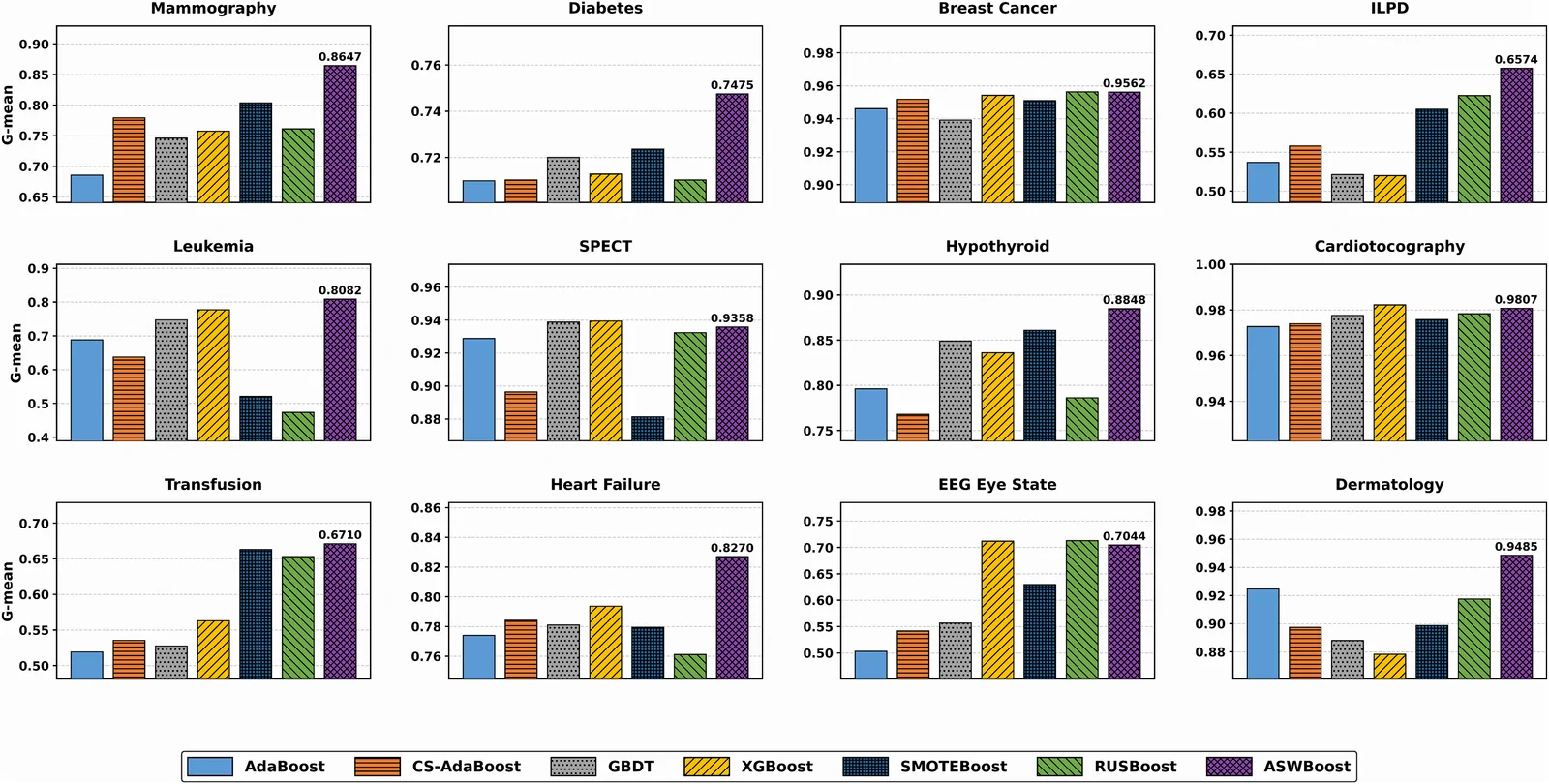
G-mean evaluation of various algorithms across 12 real-world OpenML datasets.

Fig 6 further presents the G-mean results, a metric that evaluates balanced predictive performance across both majority and minority classes.

Unlike overall accuracy, G-mean strictly penalizes models biased toward the majority class. As illustrated, ASWBoost consistently achieves the highest G-mean scores across most datasets, demonstrating particularly pronounced advantages on severely imbalanced sets such as *Mammography*, *Hypothyroid*, and *Leukemia*. By suppressing outlier interference and adaptively emphasizing hard-to-learn minority instances, ASWBoost substantially outperforms CS-AdaBoost, SMOTEBoost, and RUSBoost, while remaining highly competitive with GBDT and XGBoost. These findings confirm that the parameterized exponential loss mechanism maintains an optimal trade-off between minority-class recall and majority-class recall without artificially altering the underlying data distribution.

### 4.5. Statistical significance analysis

To rigorously evaluate the statistical significance of the performance differences among the compared algorithms across the 12 OpenML datasets, we conducted non-parametric statistical tests following the authoritative guidelines by Demšar [31]. Addressing the common limitation of relying on a single metric, this validation was comprehensively extended across all primary evaluation metrics: AUC, Balanced Accuracy, and G-mean.

As a non-parametric method, the Friedman test operates on the average ranks of the algorithms rather than their raw performance values, rendering it inherently robust to varying data distributions and incommensurate metric scales. For each metric, we ranked the k=7 algorithms on each of the N=12 datasets (assigning a rank of 1 to the best-performing algorithm and 7 to the worst, with ties receiving average ranks). The exact average ranks for all algorithms across the three metrics are explicitly summarized in Table 3.

**Table 3:** Average ranks of the compared algorithms across 12 real-world OpenML datasets.

Metric	AdaBoost	CS-AdaBoost	GBDT	XGBoost	SMOTEBoost	RUSBoost	ASWBoost
AUC	4.375	5.250	2.583	2.792	5.250	5.000	2.275
Balanced Accuracy	5.417	5.000	4.083	3.417	4.333	4.250	1.500
G-mean	5.667	4.958	4.583	3.500	3.917	3.875	1.500

The Friedman test evaluates the null hypothesis $H_{\text{0}}$ that all algorithms perform equivalently. The initial Friedman statistic $\chi_{F}^{\text{2}}$ is defined as:


$$\begin{array}{c} \chi_{F}^{\text{2}}=\frac{12N}{K(K+1)}\left[\sum_{j=1}^{K}R_{j}^{\text{2}}-\frac{K(K+1{)}^{\text{2}}}{\text{4}}\right] \end{array}$$ (21)

Because the $\chi_{F}^{\text{2}}$ statistic is known to be undesirably conservative, we utilized the corrected Iman-Davenport statistic $F_{F}$, which is distributed according to an F-distribution with K−1 and (K−1)(N−1) degrees of freedom:


$$\begin{array}{c} F_{F}=\frac{(N-1)\chi_{F}^{\text{2}}}{N(K-1)-\chi_{F}^{\text{2}}} \end{array}$$ (22)

For AUC, Balanced Accuracy, and G-mean, the computed $F_{F}$ statistics are 5.45, 5.74, and 6.67, respectively. All values significantly exceed the critical value of F(6,66)≈2.25 at the significance level α=0.05. Consequently, the null hypothesis $H_{\text{0}}$ is firmly rejected, indicating statistically significant performance differences among the algorithms across all metrics. To ascertain the specific superiorities of the proposed algorithm, we proceeded with the Bonferroni-Dunn post-hoc test [31]. This specific test is strictly appropriate for our scenario, where multiple baseline classifiers are systematically compared against a single control classifier (ASWBoost). The performance of a baseline algorithm is considered significantly different from the control algorithm if their average ranks differ by at least the Critical Difference (CD), formulated as:


$$\begin{array}{c} CD=q_{\alpha }\sqrt{\frac{K(K+1)}{6N}} \end{array}$$ (23)

Given K=7 algorithms, N=12 datasets, and a stringent significance level of α=0.05, the critical value $q_{0.05}$, yielding a rigorous CD threshold of 2.111.

Based on this mathematical threshold, we draw the following statistically backed conclusions from Table 3:

(1) For G-mean and Balanced Accuracy: ASWBoost consistently achieves the lowest average rank (1.500). The rank differences between ASWBoost and standard AdaBoost, CS-AdaBoost, GBDT, SMOTEBoost, and RUSBoost all strictly exceed the CD threshold of 2.111. This statistically confirms that ASWBoost significantly outperforms these five algorithms at α=0.05. XGBoost is the only evaluated algorithm that demonstrates statistically comparable performance to ASWBoost on these two comprehensive metrics.
(2) For AUC: ASWBoost ranks highly, aligning with advanced gradient-based frameworks (GBDT and XGBoost). Statistical testing verifies that ASWBoost significantly outperforms CS-AdaBoost, SMOTEBoost, and RUSBoost, while maintaining statistically comparable performance to standard AdaBoost, GBDT, and XGBoost.

**Fig 7:**
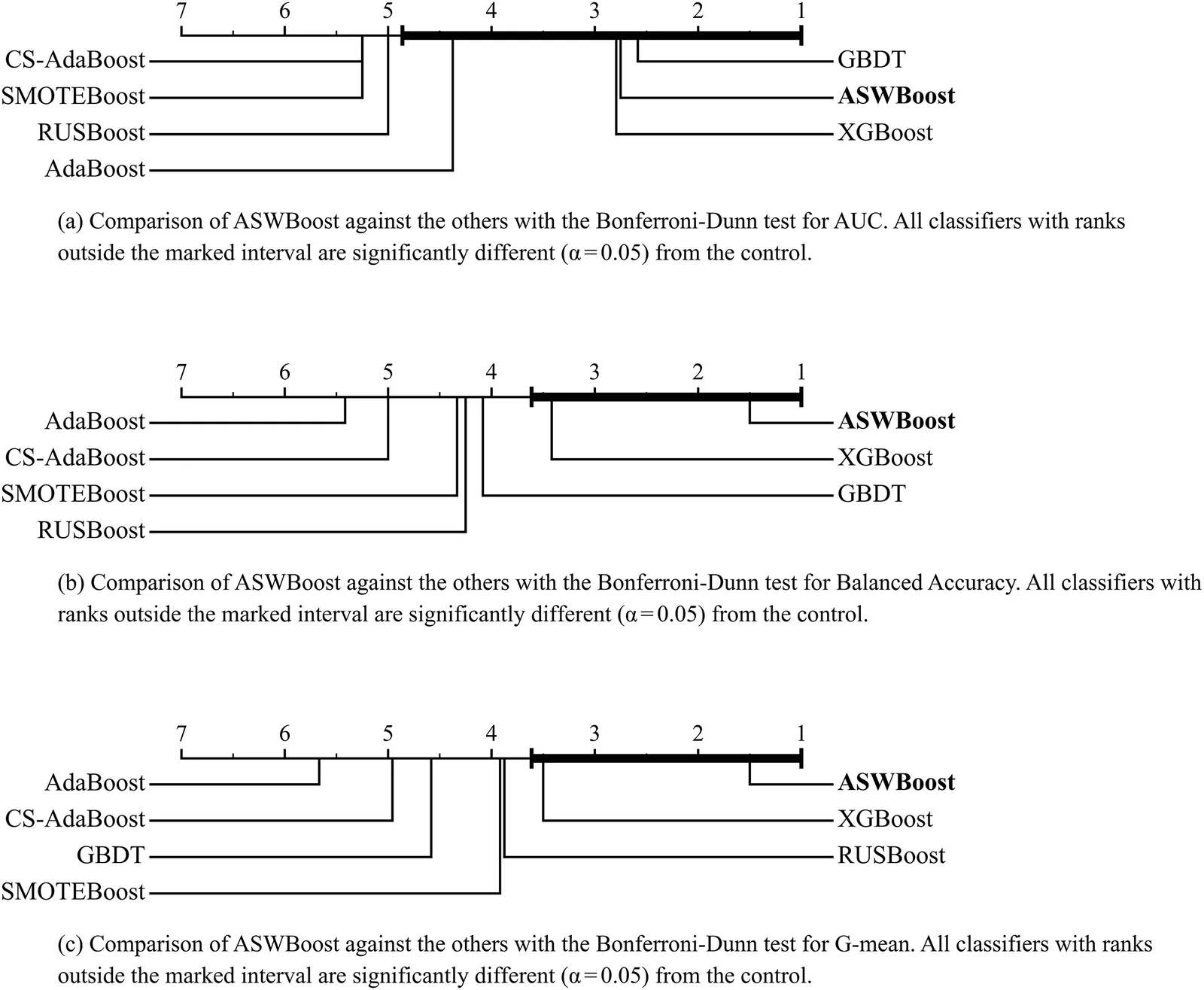
Critical difference (CD) diagrams for AUC, Balanced Accuracy, and G-mean under the Bonferroni-Dunn test (α=0.05) based on the 12 real-world OpenML datasets.

In Fig 7, the average ranks of all algorithms are plotted on the horizontal axis, with lower ranks positioned to the right. Algorithms connected by a thick solid line exhibit no statistically significant performance difference.

The analysis confirms that the performance of ASWBoost is significantly superior to AdaBoost, CS-AdaBoost, GBDT, SMOTEBoost, and RUSBoost. Although the average rank of ASWBoost is better than that of XGBoost, under the current statistical testing framework, the performance of the two algorithms is considered comparable.

## 5. Conclusion

To address the performance degradation of the AdaBoost algorithm in environments characterized by data noise and class imbalance, this paper proposes a robust boosting algorithm named ASWBoost. By introducing the parameter θ to improve the traditional exponential loss function, the algorithm achieves adaptive adjustment of sample weights while preserving the desirable properties of convex optimization. The optimal parameter for different datasets is determined via grid search. Experimental results on a synthetic dataset and 12 real-world OpenML datasets demonstrate that ASWBoost outperforms the baseline models across evaluation metrics including Balanced Accuracy, G-mean, Macro-F1, and AUC. Furthermore, the *Friedman* test and *Nemenyi post-hoc* analysis validate the statistically significant advantages of ASWBoost.

However, the determination of the current parameter relies on grid search and lacks prior data information, which leads to lower tuning efficiency and limited interpretability. Future research will explore dynamic parameter adjustment strategies based on data distribution and noise estimation. Additionally, extending ASWBoost to multi-class classification and regression tasks will be investigated to further validate the generalization capabilities of the algorithm.
