# Supplementary material for: ASWBoost: Classification algorithm for noisy and imbalanced data based on parametric exponential loss
Source: PLoS One. 2026 Aug 27;21(8):e0356889. doi: 10.1371/journal.pone.0356889 (PMC13521417; doi:10.1371/journal.pone.0356889)
Supplement: Supplementary file 1 [file pone.0356889.s001.docx]

**Reviewer Report**

Manuscript Title: *Smooth Bounded Exponential Loss Based AdaBoost for Noisy and Imbalanced Data Classification*
Manuscript ID: PONE-D-25-60888
Journal: PLOS ONE
Article Type: Research Article

General Assessment

This manuscript proposes an improved boosting algorithm (ASWBoost) by introducing a smooth bounded exponential loss function with a tunable parameter to enhance robustness against noisy and imbalanced datasets. The topic is relevant to ensemble learning and classification under real-world data imperfections and fits within the scope of *high impact factor journal*. The authors provide theoretical analysis of training error bounds and experimental validation on simulated and real datasets.

While the study shows promise, the manuscript in its current form contains significant issues related to mathematical consistency, clarity of derivations, experimental rigor, reproducibility, and presentation quality. These issues limit the reliability and interpretability of the results. Therefore, I recommend major revision before the manuscript can be considered for publication.

Major Comments

1. Novelty and Positioning Relative to Existing Work

The proposed method represents an incremental modification of AdaBoost through the introduction of a control parameter that moderates sample weight updates. Similar concepts have already been explored in the literature, including Bounded exponential loss function based AdaBoost ensemble of OCSVMs, cost-sensitive AdaBoost, AWABoost, RobustBoost, and self-paced boosting methods.

- The manuscript does not clearly articulate how ASWBoost fundamentally differs from these existing approaches.
- The contribution would be strengthened by a systematic comparison of mechanisms, explicitly highlighting what is new in the loss function design, optimization strategy, or theoretical guarantees.

Recommendation:
Include a comparative discussion or table summarizing differences between ASWBoost and closely related boosting variants, focusing on loss functions, parameter roles, noise handling, and imbalance handling.

2. Mathematical Consistency and Theoretical Soundness

Several mathematical issues require careful correction:

- αₘ is defined multiple times with conflicting formulas, e.g.:
  - Equation (2):

$$\alpha_{m}= \frac{1}{2}\log\frac{1-e_{m}}{e_{m}-\theta}$$

  - Equation (16):

$$\alpha_{m}^{*}= \frac{1}{2(1+ \theta)}\log\frac{1-e_{m}}{e_{m}}$$

These are not equivalent, yet the paper claims they are identical.

- The proposed “bounded exponential loss” is not rigorously shown to be bounded. Explicit upper and lower bounds are not provided.
- Proofs of Theorems 2 and 3 are excessively long, difficult to follow, and contain notational inconsistencies and redundant steps.

Recommendation:

- Use a single, consistent definition of αₘ and clearly derive it from loss minimization.
- Explicitly prove or demonstrate the boundedness and smoothness of the proposed loss function.
- Move lengthy derivations to an appendix and provide clearer, more concise proofs in the main text.

3. Algorithm Description and Clarity

Although the ASWBoost algorithm is described in detail, the presentation lacks clarity:

- The algorithm description repeats equations already given earlier without adding clarity.
- Algorithm 1 repeats equations already stated earlier.
- Notation switches between:
  - $G\left( x \right), f(x)$
  - $\omega_{i},D_{m}$
- The role of θ > 0 vs θ < 0 is not algorithmically explicit
- The practical interpretation of the parameter θ (positive vs. negative values) is not sufficiently illustrated.

Recommendation:

- Provide a simplified and self-contained pseudocode.
- Include a schematic or flowchart illustrating how θ affects weight updates in noisy versus imbalanced settings.

4. Experimental Design and Evaluation

4.1 Synthetic Dataset Construction

The manuscript lacks sufficient detail regarding synthetic data generation:

- Gaussian distribution parameters, noise injection mechanisms, and redundant feature construction are not fully specified.
- This omission hinders reproducibility.

4.2 Baseline Comparisons

The experimental comparison is limited primarily to AdaBoost.

- Important baselines such as Bounded exponential loss function based AdaBoost ensemble of OCSVMs, RobustBoost, cost-sensitive AdaBoost, Gradient Boosting, and modern ensemble methods (e.g., XGBoost) are missing.
- No statistical significance tests are conducted.

Recommendation:

- Include additional competitive baselines.
- Apply statistical tests (e.g., Friedman test with post-hoc analysis) to support claims of superiority.

5. Real-World Dataset and Ethical Considerations

The cardiovascular disease dataset is used as a practical application, but critical information is missing:

- Dataset source and preprocessing steps are not described.
- The validation strategy (train/test split or cross-validation) is unclear.
- No ethical approval or public-dataset statement is provided, despite involving medical data.

Recommendation:
Clearly state the dataset origin, ethical considerations, and validation methodology in compliance with PLOS ONE guidelines.

6. Reproducibility and Transparency

The manuscript does not meet reproducibility expectations:

- No code or implementation details are provided.
- Random seed control and hyperparameter ranges are not specified.
- Computational complexity and runtime comparisons are absent.

Recommendation:
Provide code availability, implementation details, and computational analysis to ensure reproducibility.

Minor Comments

1. Several references are broken or incorrectly formatted (e.g., “Error! Reference source not found”).
2. Numerous grammatical and typographical errors are present throughout the manuscript.
3. Tables and figures suffer from formatting issues and readability problems.
4. Some equations are poorly aligned and inconsistently numbered.

Recommendation:
Thorough proofreading, reference correction, and formatting cleanup are required.

Final Recommendation

Decision: Major Revision

The manuscript has potential but requires substantial revision to address mathematical correctness, experimental rigor, clarity, and reproducibility. With careful revision and expanded evaluation, the work could become a valuable contribution to robust ensemble learning research.
