# Supplementary material for: ASWBoost: Classification algorithm for noisy and imbalanced data based on parametric exponential loss
Source: PLoS One. 2026 Aug 27;21(8):e0356889. doi: 10.1371/journal.pone.0356889 (PMC13521417; doi:10.1371/journal.pone.0356889)
Supplement: Supplementary file 2 [file pone.0356889.s002.docx]

**Manuscript Draft**

Original Manuscript Title: *Smooth Bounded Exponential Loss Based AdaBoost for Noisy and Imbalanced Data Classification*

Manuscript ID: PONE-D-25-60888

Journal: PLOS ONE

Article Type: Research Article

Corresponding Author: Mei Yan

First Author: Fei Meng

Order of Authors: Fei Meng; Mei Yan; Hang Liu; Wan Xu; Hao Li

**To:** Dr. Kaywan Othman Ahmed Academic Editor *PLOS ONE*

**Journal:** PLOS ONE

**Subject:** Resubmission of Manuscript PONE-D-25-60888

March 3, 2026

Dear Professor Ahmed,

Thank you very much for your encouraging decision on our paper.

Accompanying this letter, the point-by-point responses to the AE and five referees. We hope this revision is to your satisfaction and we look forward to hearing from you soon.

Best regards,

Mei Yan

Responses to comments on manuscript:

**Responses to comments from the AE**

It was very considerate and kind of you to proofread our paper. Thank you very much for your critical and constructive comments. We have done our best to accommodate your comments and suggestions when revising our paper. For your reference, your comments are reproduced below, followed by our response.

**Title Revision**: We have revised the title to “*ASWBoost: Classification Algorithm for Noisy and Imbalanced Data Based on Parametric Exponential Loss*.” We have removed the “*Smooth Bounded”* to avoid potential confusion. This term was originally intended to refer to the training error upper bound rather than the loss function itself. We apologize for this oversight and believe the new title more accurately reflects the scope of our work.

We have carefully studied all the comments and have made substantial revisions to the manuscript accordingly, including:

1. **Novelty and Positioning Relative to Existing Work**

The proposed method represents an incremental modification of AdaBoost through the introduction of a control parameter that moderates sample weight updates. Similar concepts have already been explored in the literature, including Bounded exponential loss function based AdaBoost ensemble of OCSVMs, cost-sensitive AdaBoost, AWABoost, RobustBoost, and self-paced boosting methods.

- The manuscript does not clearly articulate how ASWBoost fundamentally differs from these existing approaches.
- The contribution would be strengthened by a systematic comparison of mechanisms, explicitly highlighting what is new in the loss function design, optimization strategy, or theoretical guarantees.

Recommendation:
Include a comparative discussion or table summarizing differences between ASWBoost and closely related boosting variants, focusing on loss functions, parameter roles, noise handling, and imbalance handling.

**Done.**

**Response**: We sincerely thank the reviewer for this insightful comment. We agree that clarifying the theoretical positioning of ASWBoost relative to existing variants is crucial for demonstrating its contribution. We have carefully revised the manuscript to explicitly distinguish ASWBoost from RobustBoost, Cost-Sensitive Boosting, SPLBoost, and AWABoost.

Although ASWBoost introduces a parameter to control weight updates, this is not merely a heuristic modification. The parameter is derived from a modified exponential loss function that fundamentally alters the weight update strategy while preserving convexity.

In the revised Introduction sections, we have added a systematic comparison discussing the mechanisms, advantages, and limitations of these methods. The key distinctions are summarized as follows:

- **vs. RobustBoost (Non-convex Loss):** RobustBoost employs a non-convex loss function based on Brownian motion, which destroys the convex optimization property of standard Boosting, leading to instability and sensitivity to hyperparameters. ASWBoost, in contrast, introduces a parameter$\theta$ into the exponential loss without truncation, preserving the convexity of the objective function. This ensures a stable global optimum and consistent convergence (as proven in our new Theorem 2).
- **vs. Cost-Sensitive AdaBoost (Fixed Costs):** Cost-sensitive methods rely on a pre-defined, fixed cost matrix. This often leads to overfitting outliers in the minority class. ASWBoost does not use a fixed matrix; instead, the parameter $\theta$ dynamically adjusts the gradient of the loss function. This allows the model to focus on hard samples without blindly increasing the weights of potential noise, offering better robustness.
- **vs. SPLBoost (Explicit Self-Paced Learning):** SPLBoost combines a classification loss with a self-paced regularization term to explicitly select easy samples, which is computationally complex. ASWBoost achieves a similar noise-filtering effect implicitly through its continuous weight adjustment mechanism. It down-weights noise naturally via the loss function curvature rather than requiring a hard/easy sample selection step, making it more computationally efficient.
- **vs. AWABoost (Heuristic Adjustment):** AWABoost modifies the weight update step heuristically by inserting a control function to limit weight growth. Its robustness comes from constraining the procedure. In contrast, ASWBoost’s weight update rule is mathematically derived directly from minimizing our proposed objective function. This ensures theoretical consistency between the loss function and the algorithm’s behavior, guaranteeing the training error bound.

We appreciate the reviewer’s suggestion regarding the OCSVM-based AdaBoost ensemble. While we acknowledge its relevance to robust learning, we did not include it in our comparison for two primary reasons. First, to ensure a fair evaluation of the boosting framework itself, we maintained consistency by using decision stumps as the base learner for all methods. Introducing an SVM-based ensemble would make it difficult to discern whether performance variations arise from the loss function or the inherent difference in inductive bias between trees and SVMs. Second, OCSVM-based methods are typically tailored for one-class or anomaly detection tasks, whereas ASWBoost is designed for supervised binary classification under noise and imbalance. We appreciate the pointer and will consider exploring OCSVM-based ensembles in our future research.

2. **Mathematical Consistency and Theoretical Soundness**

Several mathematical issues require careful correction:

- αₘ is defined multiple times with conflicting formulas, e.g.:
  - Equation (2):

$$\alpha_{m}= \frac{1}{2}\log\frac{1-e_{m}}{e_{m}-\theta}$$

  - Equation (16):

$$\alpha_{m}^{*}= \frac{1}{2(1+ \theta)}\log\frac{1-e_{m}}{e_{m}}$$

These are not equivalent, yet the paper claims they are identical.

- The proposed “bounded exponential loss” is not rigorously shown to be bounded. Explicit upper and lower bounds are not provided.
- Proofs of Theorems 2 and 3 are excessively long, difficult to follow, and contain notational inconsistencies and redundant steps.

Recommendation:

- Use a single, consistent definition of αₘ and clearly derive it from loss minimization.
- Explicitly prove or demonstrate the boundedness and smoothness of the proposed loss function.
- Move lengthy derivations to an appendix and provide clearer, more concise proofs in the main text.

**Done.**

**Response**: Thank you for this important observation. We have re-examined the mathematical derivations and thoroughly revised Section 3 to ensure consistency and rigor. In particular, by minimizing the objective function (Eq. 11) using Fermat’s principle, we derive the optimal coefficient $\alpha_{m}$. As presented in the revised Section 3.1 (Eq. 15) and Algorithm 2, $\alpha_{m}$ is given uniformly by

$$\alpha^{*}=\frac{1}{2+\theta}\ln\frac{1-e_{m}}{(1+\theta)e_{m}}$$

We agree with the reviewer’s assessment that the term ‘Bounded’ was imprecise, as the loss function is convex and smooth but not strictly bounded in a mathematical sense. Consequently, we have revised the manuscript title to “*ASWBoost: Classification Algorithm for Noisy and Imbalanced Data Based on Parametric Exponential Loss*.” Furthermore, we have standardized the mathematical notation throughout the paper, provided a rigorous proof for the training error bounds, and streamlined the derivation steps for improved clarity. As shown in Figure 1, the smoothness of our proposed loss function is now clearly illustrated.

**3. Algorithm Description and Clarity**

Although the ASWBoost algorithm is described in detail, the presentation lacks clarity:

- The algorithm description repeats equations already given earlier without adding clarity.
- Algorithm 1 repeats equations already stated earlier.
- Notation switches between:
  - $G\left( x \right), f(x)$
  - $\omega_{i},D_{m}$
- The role of θ > 0 vs θ < 0 is not algorithmically explicit
- The practical interpretation of the parameter θ (positive vs. negative values) is not sufficiently illustrated.

Recommendation:

- Provide a simplified and self-contained pseudocode.
- Include a schematic or flowchart illustrating how θ affects weight updates in noisy versus imbalanced settings.

**Done.**

**Response**: To address these issues, we have comprehensively revised Section 3 and Algorithm 2. The specific improvements are as follows:

- Simplified Pseudocode: As recommended, We retained Algorithm 1 to outline standard AdaBoost for baseline context, and we have provided a simplified, self-contained pseudocode for our proposed method in Algorithm 2.
- Unified Notation: We have thoroughly proofread the manuscript and standardized all mathematical notations. We now consistently use $G_{m}(x)$ to denote the base classifier and $D_{m}$ to represent the weight distribution, eliminating the confusion between mixed notations like $f(x)$ or $\omega_{i}$.
- Visual Illustration of $\theta$: To clarify the practical interpretation of the parameter $\theta$, we have added a new schematic diagram, Figure 1 (Section 3.3), and expanded the corresponding discussion.
- We explicitly explain that when $\theta>0$, the algorithm imposes a larger penalty on misclassified samples, thereby increasing the focus on the minority class (Imbalance Handling).
- Conversely, when $\theta<0$, the weight update becomes more conservative to suppress the influence of outliers, enhancing robustness against label noise (Noise Handling).

**4. Experimental Design and Evaluation**

**4.1 Synthetic Dataset Construction**

The manuscript lacks sufficient detail regarding synthetic data generation:

- Gaussian distribution parameters, noise injection mechanisms, and redundant feature construction are not fully specified.
- This omission hinders reproducibility.

**4.2 Baseline Comparisons**

The experimental comparison is limited primarily to AdaBoost.

- Important baselines such as Bounded exponential loss function based AdaBoost ensemble of OCSVMs, RobustBoost, cost-sensitive AdaBoost, Gradient Boosting, and modern ensemble methods (e.g., XGBoost) are missing.
- No statistical significance tests are conducted.

Recommendation:

- Include additional competitive baselines.
- Apply statistical tests (e.g., Friedman test with post-hoc analysis) to support claims of superiority.

**Done.**

**Response:** In response to this comment, we have completely rewritten the experimental section (Section 4). Our specific revisions are as follows:

**1.**  Regarding Synthetic Dataset Construction (Section 4.2) We have provided a detailed description of the synthetic data generation process to ensure reproducibility. Specifically:

- Data Structure:we have utilized the make_classification tool to generate a synthetic dataset consisting of 1,000 samples and 40 features.
- Feature Generation: We specified that 25 informative features are generated using Gaussian clusters with a standard deviation of 1.0 and a class separation factor of 1.5. Additionally, 10 redundant features are linear combinations of the informative ones, and 5 noise features are drawn from a standard normal distribution N(0,1).
- Noise Injection: We explicitly stated that label noise was simulated by randomly flipping class labels at rates of 10%, 20%, and 30%.

**2.** Regarding Baseline Comparisons (Section 4.2 & 4.4) We have significantly expanded our comparative analysis. Instead of limiting the comparison to AdaBoost, we have introduced a comprehensive set of state-of-the-art and classic baselines:

- Robustness Baselines: We added RobustBoost and Cost-Sensitive AdaBoost (CS-AdaBoost) to evaluate performance under noise and imbalance.
- Advanced Ensemble Methods: We included GBDT and XGBoost as strong baselines to test the competitiveness of ASWBoost against modern gradient boosting frameworks.
- Imbalance Handling Baselines: For the real-world datasets, we also compared against SMOTEBoost (oversampling) and RUSBoost (undersampling) to validate our re-weighting strategy.

3. Regarding Statistical Significance Tests (Section 4.4) To ensure the reliability of our conclusions, we have incorporated rigorous statistical testing. We employed the Friedman test to detect statistical differences among the algorithms across the 12 OpenML datasets. Upon rejecting the null hypothesis ($p<0.05$), we conducted the Nemenyi post-hoc test to identify specific pairwise differences. The critical difference (CD) diagrams have been added as Figure 6 to visually present these results.

**5. Real-World Dataset and Ethical Considerations**

The cardiovascular disease dataset is used as a practical application, but critical information is missing:

- Dataset source and preprocessing steps are not described.
- The validation strategy (train/test split or cross-validation) is unclear.
- No ethical approval or public-dataset statement is provided, despite involving medical data.

Recommendation:
Clearly state the dataset origin, ethical considerations, and validation methodology in compliance with PLOS ONE guidelines.

**Done.**

**Response**: Thank you for pointing out these critical omissions. We fully agree that transparency regarding data sources, preprocessing, and ethical compliance is essential. we have made the following substantial revisions in Section 4.4 (OpenML Datasets) and Table 2 of the revised manuscript:

- Dataset Source and Expansion: We have replaced the single cardiovascular dataset with 12 publicly available benchmark datasets from the OpenML repository (**https://www.openml.org/**), which includes multiple medical datasets (e.g., Heart Failure, Mammography, Diabetes). Detailed statistics for these datasets are now listed in Table 2.
- Preprocessing Details: We have explicitly described the data preprocessing steps. As stated in Section 4.4, all features were standardized to have a mean of 0 and a standard deviation of 1 to ensure consistent model training.
- Validation Strategy: We have clarified our validation methodology. To ensure robust performance evaluation, we employed a stratified 5-fold cross-validation strategy for all experiments, as detailed in Section 4.1 and Section 4.4.
- Ethical Considerations: We have included a statement regarding ethical compliance. Since this study exclusively utilizes publicly available, anonymized datasets from the OpenML repository, no specific ethical approval or patient consent was required.

**6. Reproducibility and Transparency**

The manuscript does not meet reproducibility expectations:

- No code or implementation details are provided.
- Random seed control and hyperparameter ranges are not specified.
- Computational complexity and runtime comparisons are absent.

Recommendation:
Provide code availability, implementation details, and computational analysis to ensure reproducibility.

**Done.**

**Response**: We have addressed this comment by making the following substantial revisions:

**1. Open Source Code:** We have uploaded the complete source code, including the implementation of the ASWBoost algorithm and the scripts for reproducing the experiments, to a public GitHub repository.

- Repository Link: <https://github.com/064213mf/ASWBoost.git>
- This link has also been added to the code availability

**2. Implementation Details & Random Seed:** To ensure result reproducibility, we have explicitly detailed the experimental setup in Section 4 (Experimental Design and Evaluation):

- **Platform:** All algorithms were implemented using Python 3.8 and the Scikit-learn library.
- **Random Seed:** We standardized the random seed to 42 for all data splitting, model initialization, and noise generation processes to guarantee that all results are deterministic and reproducible.
- **Validation Strategy:** We employed a 5-fold stratified cross-validation for the outer loop to evaluate performance, ensuring class distribution consistency across folds.

**3. Hyperparameter Specifications: We have clarified the parameter settings in Section 4.4:**

- **Proposed Method (ASWBoost):** The smoothing parameter $\theta$ was tuned via grid search within the range of $(-1,1)$. The number of boosting iterations$(M)$ was set to 50.
- **Baselines:** All comparison models (AdaBoost, Gradient Boosting, XGBoost, etc.) were constructed using Scikit-learn or their respective standard libraries with default parameters unless otherwise specified, with the iteration count uniformly set to 50 for fair comparison.
- **Complexity and Runtime Analysis:** We have added a new subsection, Section 4.3 Complexity Analysis, to the revised manuscript.
- **Theoretical Analysis:** We demonstrated that ASWBoost maintains the same computational complexity order as standard AdaBoost, which is $O(M\cdot N\cdot d)$, where N is the sample size and d is the feature dimension.
- **Empirical Comparison:** We added Table 1, which records the actual running times of ASWBoost against five other baseline algorithms on synthetic datasets. While ASWBoost requires slightly more time due to the parameter tuning process, the analysis confirms its computational feasibility for practical applications.

**Minor Comments**

1. Several references are broken or incorrectly formatted (e.g., “Error! Reference source not found”).
2. Numerous grammatical and typographical errors are present throughout the manuscript.
3. Tables and figures suffer from formatting issues and readability problems.
4. Some equations are poorly aligned and inconsistently numbered.

Recommendation:
Thorough proofreading, reference correction, and formatting cleanup are required.

**Done.**

**Comment 1:** Several references are broken or incorrectly formatted (e.g., “Error! Reference source not found”).

**Response:** Thank you for pointing this out. We have thoroughly checked the entire manuscript and corrected all citation errors. All broken links and “Error! Reference source not found” messages have been resolved. We have also verified that the reference list strictly follows the journal’s formatting guidelines.

**Comment 2:** Numerous grammatical and typographical errors are present throughout the manuscript.

**Response:** We apologize for the grammatical and typographical errors in the original submission. We have carefully proofread the revised manuscript to correct these issues. Additionally, we have standardized the terminology to ensure clarity and professional presentation throughout the text.

**Comment 3:** Tables and figures suffer from formatting issues and readability problems.

**Response:** We have redrawn and reformatted the figures and tables to improve their quality and readability. Specifically:

- **Figures:** We have updated the figures (e.g., Figure 1, Figure 2, etc.) with higher resolution and clearer legends to ensure they are self-explanatory.
- **Tables:** We have reformatted the tables (e.g., Algorithm 1, Algorithm 2, Table 1, Table 2) to ensure proper alignment and consistent style.

**Comment 4:** Some equations are poorly aligned and inconsistently numbered.

**Response:** We have reviewed all mathematical expressions in the manuscript. All equations have been re-typed and aligned properly using standard mathematical editors. We have also ensured that equation numbering is consecutive and consistent throughout the paper.

**Comment 5**: We note you have included a table to which you do not refer in the text of your manuscript. Please ensure that you refer to Table 4 & 5 in your text; if accepted, production will need this reference to link the reader to the Table.

**Response**: We sincerely thank the Editor for pointing this out. During this major revision, we completely restructured the Experimental section (Section 4) to address the reviewers’ comments. Specifically, we expanded our evaluation from a single dataset to 12 diverse OpenML datasets and incorporated comprehensive statistical significance tests (Friedman and Nemenyi tests).

As a result of this extensive restructuring, the presentation of our results has been entirely overhauled. The data previously contained in the original Tables 4 and 5 has been removed or consolidated. Our revised manuscript now contains exactly three tables:

Table 1: Computational time comparison on synthetic datasets.

Table 2: Characteristics of the 12 OpenML datasets.

Table 3: G-mean values of various algorithms on OpenML datasets.

We have carefully verified that all three of these current tables are properly cited and referred to within the main text of the revised manuscript. We apologize for any confusion caused by the residual tables in the previous draft.

We have made every effort to address the reviewer’s comments meticulously. The revised manuscript now features a more rigorous theoretical framework, a transparent algorithm description, and comprehensive experimental comparisons against state-of-the-art baselines. We appreciate your renewed consideration of our work and look forward to your positive response.

Yours sincerely,

Mei Yan

*Detailed Response to Reviewers

Responses to comments on manuscript PONE-D-25-60888

**Responses to comments from Reviewer #1**

We would like to express our sincere gratitude to you for your positive comments regarding the technical depth and quality of our work. Following your constructive suggestions, we have carefully implemented the recommended improvements throughout the revised manuscript.

- The manuscript would benefit from a more explicitly defined problem statement. While the need for the research can be inferred, the authors should clearly articulate the problem being addressed, including not only the algorithmic improvement but also the rationale for its application to the selected dataset and practical use case.

**Point taken.**

**Response:** Thank you for this suggestion. We have revised the Introduction to. explicitly define the problem statement. We clarified that while AdaBoost has strong generalization abilities, it is highly sensitive to noisy data and class imbalance, which leads to overfitting and performance degradation. We have articulated the rationale for developing ASWBoost: to enhance robustness by introducing a parameter that modify the traditional exponential loss function, thereby improving performance in complex data environments.

- The opening sentence of the introduction, “When strong learners are difficult to learn directly in machine learning, Boosting provides an effective design framework for ensemble learning,” should be revised. As the first sentence of an academic manuscript, it should reflect a more polished scholarly tone, avoid awkward phrasing, and clearly establish context with sufficient technical depth. In addition, the introductory paragraph would benefit from improved logical flow and cohesion, as statements such as “AdaBoost has good generalization ability and is not prone to overfitting” appear somewhat isolated without clearer linkage to preceding sentences.

**Done.**

**Response:** We have rewritten the opening sentence and the introductory. paragraph to improve the scholarly tone and logical flow. The revised introduction now begins: *“Within the framework of Boosting ensemble learning, AdaBoost can boost weak classifiers into a strong classifier without prior knowledge. Its superior classification performance has attracted widespread attention in the machine learning community. This ensemble mechanism provides AdaBoost with robust generalization capabilities, demonstrating highly competitive classification performance across various critical domains, including text classification, feature selection, object recognition, spam filtering and face recognition.”* and proceeds to logically connect AdaBoost’s mechanism with its limitations in noisy environments, ensuring better cohesion between sentences.

- The repeated use of the phrase “In practical applications” throughout the introduction is stylistically repetitive and should be reviewed. Similarly, the frequent use of “the AdaBoost” is, in many instances, grammatically unnecessary and overly repetitive and should be corrected to align with standard academic usage.

**Done.**

**Response:** We have carefully proofread the manuscript to remove repetitive phrases such as “In practical applications” and corrected the grammatical usage of “AdaBoost” (removing the unnecessary article “the”) throughout the text to align with standard academic usage.

- In the sentence “This mechanism forces subsequent weak classifiers to overly focus on noisy samples, thereby seriously reducing the learning performance of the model,” the expressions “overly focus” and “seriously reducing” appear subjective and insufficiently objective for academic writing. More measured and precise phrasing is recommended. In addition, multiple instances of “[Error! Reference source not found.]” should be corrected.

**Done.**

**Response:** We have replaced these subjective expressions with more objective and technical terminology. revise: “ *However, since the AdaBoost algorithm continuously increases the weight of misclassified samples during iterations, in noisy datasets, samples that are difficult to classify correctly are often the noise itself. This readily leads to the model overfitting the noisy data.* “ We have fixed all broken cross-references and citation errors in the manuscript.

- The paragraph outlining the paper structure (e.g., “The structure of this paper is as follows…”) does not appear necessary in the introduction of an academic research article and should be reviewed, particularly if not required by the journal’s formatting guidelines.

**Response:** Have deleted

- The authors should consider reorganising the overall manuscript structure and adopting more professional and standardised chapter or section headings. In particular, clearer separation between the background, the proposed methodology, theoretical analysis, experimental evaluation, results, and conclusion would significantly improve readability and accessibility for a broad audience.

**Done.**

**Response:** We have completely reorganized the manuscript structure. The paper now follows a standard academic structure with clear section headings: 1 Introduction, 2 The proposed methodology, 3 Theoretical Analysis of ASWBoost, 4 Experimental Results and Analysis, and 5 Conclusion.

- From a methodological perspective, the authors should clarify whether alternative feature selection algorithms were considered and provide a brief justification for the selection of the Boruta algorithm, including the criteria or comparative considerations that informed this choice.

**Response:** This part has been deleted in the revised manuscript.

- The authors should also consider including the URL link to the Statlog dataset utilised and provide an overview of the dataset background, including a clear rationale for its selection in the context of the study.

**Point taken.**

**Response:** We have addressed this by utilizing 12 datasets from the **OpenML** repository to ensure reproducibility and diversity. We have provided the URL (<https://www.openml.org>) and a detailed table (Table 2) listing the sample size, feature number, and imbalance ratio for each dataset.

- The presentation of the evaluation results would benefit from improvement. Specifically, the authors should consider utilising a consistent table orientation aligned with the main text, as the current presentation disrupts readability and makes comparison of results less intuitive.

**Done.**

**Response:** We have reformatted the results section. Tables are now presented with consistent orientation.

- In addition, figures directly supporting the reported results should be presented within the relevant results or discussion sections, rather than being indexed at the later end of the manuscript, as this would improve interpretability and allow readers to more easily relate visual evidence to the accompanying analysis.

**Done.**

**Response:** all figures (e.g., Figure 2, Figure 3, Figure 4) are now placed immediately following their relevant analysis in the text to improve readability and interpretability.

- The concluding recommendations “Furthermore, based on the research findings, recommendations for the prevention of cardiovascular diseases ... early intervention and reducing the risk of disease onset” should be strengthened by explicitly anchoring them to the model outputs and feature-level evidence derived from the analysis.

**Done.**

**Response:** We have revised the Conclusion. We removed the generalized medical recommendations (such as cardiovascular disease prevention) that were not directly supported by our specific feature analysis. Instead, the conclusion now strictly summarizes the algorithmic performance, the effect of the parameter$\theta$, and the model’s robustness in noisy and imbalanced scenarios .

- Finally, the authors should note that the abstract should also reflect these improvements, such that it effectively conveys its intended purpose of providing a comprehensive and coherent overview of the study.

**Done.**

**Response:** We have rewritten the Abstract to provide a comprehensive and coherent overview of the study. It now clearly summarizes the theoretical reconstruction of the exponential loss function, the derivation of weight formulas, the proof of the error bound, and the empirical results on both synthetic and OpenML datasets.

We would like to express our sincere gratitude to you for the constructive feedback, which has helped us significantly improve the quality, clarity, and rigor of our manuscript.

**Responses to comments from Reviewer #2**

We would like to express our sincere gratitude to you for the constructive and insightful comments. These suggestions, particularly regarding the comparison with related work and the of experimental results, have significantly helped us improve the quality, clarity, and rigor of our manuscript. We have carefully studied the recommended articles and revised the manuscript accordingly.

- This paper presents a novel method to improve the performance of AdaBoost in environments with noisy and imbalanced data. The paper claims novelty in improving AdaBoost; however, the authors should compare their proposed method with two similar studies listed below:

1. A noise-detection based AdaBoost algorithm for mislabeled data.
2. Bounded exponential loss function based AdaBoost ensemble of OCSVMs.
If the proposed method is similar to those presented in the two above-mentioned articles, the authors should clearly explain the similarities and differences.
In addition, the experimental results section should be improved. For example, the authors should describe the dataset in detail and explain the nature of the noise and class imbalance present in the data. To further improve the clarity of the paper, the authors are encouraged to review the two above-mentioned articles and follow their presentation structure. The results should be presented using figures and charts to enhance clarity. Furthermore, the quality of the figures should be improved.

**Done.**

**Response:**

**1.** We have carefully reviewed the recommended articles and included them in our references. In the revised **Introduction**, we have added a discussion comparing our ASWBoost algorithm with these and other robust boosting methods (such as RobustBoost, SPLBoost, and Cost-Sensitive AdaBoost).

While the suggested studies also aim to handle noise, our method differs in the following key aspects:

- **Optimization Approach:** Unlike noise-detection methods that explicitly filter out samples, ASWBoost uses exponential loss function. We introduce a tunable parameter $\theta$to adjust the curvature of the traditional exponential loss function, which naturally suppresses the weight growth of noisy samples during the iterative process without discarding data explicitly.
- **Convexity:** Some bounded loss methods result in non-convex optimization problems. ASWBoost retains the convex properties of the exponential loss (to a certain extent) by using the parameter $\theta$ to control the penalty intensity rather than strictly truncating the loss, ensuring better convergence stability.
- **Theoretical Bounds:** We have provided a rigorous derivation of the training error upper bound for our specific loss function, proving that the training error still decreases exponentially.

We omitted OCSVM-based ensembles for two reasons: (1) we fixed decision stumps as the base learner to fairly evaluate the boosting framework; mixing in SVMs would confound inductive-bias differences with loss-function effects, and (2) OCSVMs are mainly for one-class/anomaly detection, whereas ASWBoost targets supervised noisy, imbalanced binary classification. We will consider OCSVM ensembles in future work.

**2.** We have significantly expanded the Experimental Section (Section 4) to provide a detailed description of both the synthetic and real-world datasets:

- **Synthetic Dataset Details:** In Section 4.2, we detailed the generation process using the make_classification tool. We explained that the dataset contains 1000 samples with 40 features (including redundant and noise features). We also explicitly described how noise was introduced by randomly flipping 10%, 20%, and 30% of the class labels to simulate label noise.
- **Real-world Datasets:** In Section 4.4, we provided Table 2, which lists the statistical information for 12 OpenML datasets, including sample size, feature dimension, and specific imbalance ratios (ranging from 1.68:1 to 42.01:1). We also explained the preprocessing steps, such as normalization and how multi-class datasets were converted to binary problems.

**3.**  We have reorganized the paper to follow a more standard and logical presentation structure found in high-quality ensemble learning literature:

**Introduction:** Clearly states the problem of noise and imbalance in AdaBoost.

**Related Work:** Systematically reviews existing solutions.

**Methodology:** Presents the theoretical derivation of ASWBoost, starting from the loss function to the algorithm implementation (Algorithm 1 & 2) and error bound proofs.

**Experiments:** Divided into subsections for Evaluation Metrics, Synthetic Data Analysis, Complexity Analysis, and Real-world Data Analysis.

**Conclusion:** Summarizes findings and future work.

**4.** We have revised the results section to rely more heavily on visual representations rather than just text or dense tables.

**5.** We have redrawn all figures (Figures 1 through 6) to ensure they meet high-resolution standards (300 dpi or vector graphics where applicable). We ensured that axis labels, legends, and data points are clearly legible and distinguishable.

**Responses to comments from Reviewer #3**

We would like to express our sincere gratitude for your professional and constructive assessment of our manuscript. We are encouraged by your recognition of the technical soundness and data availability of our work. Regarding the concerns you raised about the rigor of the statistical analysis, we have taken your feedback very seriously and have performed a thorough revision to strengthen this aspect.

Our specific responses and the corresponding revisions are as follows:

**1.** The proposed ASWBoost incorporates the parameter “theta” into the classifier weight calculation, as presented in Equations (2), (4), and (5). The authors have provided the general motivation and mechanism of using theta to control classifier weights. However, it would be beneficial to include a more intuitive explanation of how theta in Equations (2), (4), and (5) affects the smoothness of both classifier weights and sample weights. Such an explanation would help readers better understand the technical role of theta and how it influences the learning process.

**Point taken.**

**Response:** We have added a detailed explanation in Section 3.3 (ASWBoost algorithm description). Specifically, we clarified the mechanism of $\theta$: when $\theta>0$, the algorithm increases the weight update amplitude for misclassified samples, focusing more on minority classes; when $\theta<0$, the weight update becomes more conservative, effectively suppressing the impact of noise. Figure 1 has also been provided to visualize how $\theta$ influences the weight update curve.

**2.** The authors have presented all the required equations for ASWBoost in the manuscript. Therefore, I suggest simplifying the notation in Algorithm 1 by removing the explicit equations and instead referring to the corresponding equations introduced earlier in the paper. Repeating the same equations within the algorithm is unnecessary and may reduce clarity. In addition, it is unclear whether Equations (8) and (9) are part of the ASWBoost procedure described in Algorithm 1. If they are, Algorithm 1 should be revised accordingly to explicitly include these equations.

**Done.**

**Response:**

We have revised the algorithm description (now Algorithm 2) to improve clarity. The steps now correspond directly to the derived formulas. We also clarified in Section 3.1 that the optimization target (related to original Eq. 8 and 9) is used to derive the optimal parameters $\alpha_{m}$ and sample weights $\omega_{m+1}$, ensuring the logical consistency between the derivation and the algorithm steps.

**3.** The proposed method has been evaluated extensively using synthetic datasets with varying sample sizes and noise levels. However, the study lacks evaluation on real-world datasets, particularly publicly available benchmark datasets commonly used in class imbalance classification research. Real-world datasets often exhibit more complex characteristics, such as irregular data distributions, uncorrelated or redundant features, high dimensionality, severe class overlap, and the presence of small disjuncts. In my view, evaluating the proposed method solely on synthetic datasets is not sufficient when many public benchmark datasets are readily available. Therefore, I strongly recommend that the authors consider evaluating their method on real-world benchmark datasets and refer to the following relevant studies:
a. Azal Ahmad Khan, Omkar Chaudhari, Rohitash Chandra, “A review of ensemble learning and data augmentation models for class imbalanced problems: Combination, implementation and evaluation,” Expert Systems with Applications, Volume 244, 2024. https://doi.org/10.1016/j.eswa.2023.122778.
b. Fachrie, M., Musdholifah, A., and Pulungan, R., “Effectiveness of data resampling and ensemble learning in multiclass imbalance learning,” Artificial Intelligence Review, Volume 58, 368 (2025). <https://doi.org/10.1007/s10462-025-11357-w>

**Done.**

**Response:** We agree with this recommendation. In the revised manuscript, we have added 12 real-world medical datasets from OpenML (e.g., Mammography, Diabetes, Leukemia) to evaluate the algorithm’s performance in complex scenarios. The detailed statistics of these datasets are listed in Table 2. Experimental results in Section 4.4 demonstrate that ASWBoost performs well on real-world data with varying imbalance ratios and noise levels. We have reviewed and cited the recommended papers to strengthen our discussion on ensemble learning for imbalanced problems.

**4.** Since the paper focuses on handling class imbalance, I recommend evaluating the classification performance of ASWBoost using metrics that are more appropriate for imbalanced learning, such as Average Accuracy (AvAcc), Geometric Mean (G-mean), macro F1-score, and AUC. Metrics such as accuracy, precision, recall, and standard F1-score are often misleading in imbalanced classification settings. Please also provide clear and correct mathematical definitions for all evaluation metrics used in this study.

**Done.**

**Response:** We have adopted Balanced Accuracy, G-mean, Macro-F1, and AUC as the primary evaluation metrics, as they are more suitable for imbalanced tasks. The mathematical definitions for these metrics have been explicitly provided in Section 4.1.

**5.** As theta is the key parameter that plays a significant role in ASWBoost, it is necessary to provide an in-depth analysis of how different values of theta influence classification performance. I suggest conducting additional experiments using a range of theta values and presenting a comprehensive analysis of the relationship between theta and the performance of ASWBoost.

**Done.**

**Response:** We have conducted an analysis of $\theta$. Section 3.3 discusses the theoretical impact of $\theta$ values (positive vs. negative). Furthermore, in the experimental section (Section 4.2 and 4.4), we utilized grid search to determine the optimal $\theta$ for different datasets and analyzed how adjusting $\theta$ helps the model trade off between noise robustness and minority class recognition.

**6.** Please include appropriate statistical significance analysis, for example, using the Friedman test followed by a Nemenyi post-hoc test, to assess whether the performance differences between ASWBoost and other boosting algorithms are statistically significant.

**Done.**

**Response:** We have performed statistical significance tests as suggested. We utilized the Friedman test to confirm significant differences among algorithms and the Nemenyi post-hoc test for pairwise comparisons. The results are reported in Section 4.4, including the critical difference diagram in Figure 6, which confirms that ASWBoost achieves a statistically significant improvement over AdaBoost and other baselines.

**7.** Several entries in Tables 2, 3, and 4 are confusing, particularly the terms “200 datasets,” “3000 datasets,” and “10000 datasets.” It is unclear how experiments could be conducted using such a large number of datasets. I assume that the authors actually mean “200 instances,” “3000 instances,” and “10000 instances.” Please revise these tables to provide clear and accurate information.

**Done.**

**Response:**

We apologize for the confusion. We have corrected the terminology in all tables. now correctly labels the columns as “Sample Number” and “Feature Number” instead of “datasets,” ensuring accurate information presentation.

**8.** Several citations in the manuscript are not properly recognized or formatted. Please carefully review and correct all references to ensure accuracy and consistency.

**Point taken.**

**Response:** We have carefully checked and reformatted all references in the References section to ensure accuracy and consistency with the journal’s style.

**9.** Finally, the conclusion should clearly discuss the limitations of the proposed method. As no method is without limitations, this discussion is essential for providing a balanced perspective. In addition, please outline potential directions for future work that could help other researchers extend or improve upon the current study.

**Done.**

**Response:** We have expanded Section 5 (Conclusion) to include a discussion on limitations and future work. We acknowledged that the current dependence on grid search for $\theta$ reduces computational efficiency and interpretability. For future work, we proposed exploring dynamic weight adjustment strategies based on data distribution and extending the algorithm to multi-class and regression tasks.

**Responses to comments from Reviewer #4**

We sincerely thank the reviewer for the positive assessment of our topic and the constructive criticisms regarding the mathematical rigor, experimental details, and discussion depth. We have carefully revised the manuscript to address these issues.

The manuscript presents a modified AdaBoost variant (ASWBoost) using a smooth bounded exponential loss to improve robustness against noise and class imbalance. The topic is relevant and suitable for high impact factor journal, and the idea of controlling sample weight updates with a tunable parameter is meaningful. However, the paper currently suffers from mathematical inconsistencies, unclear derivations, missing experimental details, depth discuss of the experimental result, formatting errors, and insufficient comparison rigor, which must be addressed before the work can be considered for publication.

**Comment 1:** The paper currently suffers from mathematical inconsistencies and unclear derivations.

**Response:** We have rewritten Section 3 to ensure mathematical rigor and clarity.

- **Re-derivation:** We rigorously derived the ASWBoost algorithm from the Forward Stagewise Additive Modeling framework. We explicitly derived the optimal base classifier weight $(\alpha_{m})$ and the sample weight update formula (Equations 10-18).
- **Theoretical Proofs:** We added Theorem 2 and Theorem 3 to strictly prove the upper bound of the training error and analyze the convergence properties of the algorithm compared to traditional AdaBoost (Section 3.2). The mathematical inconsistencies have been resolved.

**Comment 2:** Missing experimental details.

**Response:** We have supplemented the missing details in Section 4 to ensure reproducibility:

- **Data Generation:** In Section 4.2, we detailed the generation process of the synthetic dataset, including the number of samples (1000), features (40), and the specific method for introducing noise and redundancy (using make_classification).
- **Parameter Settings:** We clarified the parameter $\theta$ settings for all algorithms. For ASWBoost, we specified the grid search range for $\theta\in(-1,1)$ and the use of 5-fold cross-validation (Section 4.4).
- **Metrics:** We added Section 4.1 to provide mathematical definitions for all evaluation metrics used: Balanced Accuracy, G-mean, Macro-F1, and AUC.

**Comment 3: Depth discuss of the experimental result.**

**Response:** We have significantly deepened the discussion of the results:

- **Mechanism Analysis:** We analyzed how the parameter $\theta$ affects the weight updates to suppress noise and handle class imbalance (Section 3.3 and Figure 1).
- **Performance Analysis:** In Section 4.2 (Synthetic Data) and Section 4.4 (OpenML Data), we analyzed *why* ASWBoost outperforms baselines like AdaBoost and RobustBoost. We discussed the specific behavior of the algorithm on high-noise and high-imbalance datasets (e.g., Mammography, Leukemia), explaining that the improvement stems from the adaptive margin control provided by $\theta$.

**Comment 4: Insufficient comparison rigor.**

**Response:** We have enhanced the rigor of our comparative analysis:

- **Statistical Tests:** We introduced the Friedman test and Nemenyi post-hoc test in Section 4.4 (Table 3 and Figure 6) to demonstrate that the performance improvements of ASWBoost are statistically significant.
- **Stronger Baselines:** We expanded the comparison to include state-of-the-art ensemble methods, including XGBoost, GBDT, SMOTEBoost, and RUSBoost, alongside the original AdaBoost variants (CS-AdaBoost, RobustBoost).

**Comment 5: Formatting errors.**

**Response:** We have thoroughly proofread the manuscript. We fixed the citation format, standardized the equation numbering, and corrected typographical errors throughout the text to meet PLOS ONE’s publication standards.

**Responses to comments from Reviewer #5**

**Comment** ：The paper discussed the enhanced version of AdaBoost that efficiently leverages imbalanced and noisy data. The author utilized real-world and benchmark datasets to prove their algorithm’s performance, which outperforms AdaBoost.

**Response:** We would like to express our sincere gratitude to you for the positive evaluation and the time spent reviewing our manuscript. We appreciate your recognition of the value of our work.
