# Supplementary material for: ASWBoost: Classification algorithm for noisy and imbalanced data based on parametric exponential loss
Source: PLoS One. 2026 Aug 27;21(8):e0356889. doi: 10.1371/journal.pone.0356889 (PMC13521417; doi:10.1371/journal.pone.0356889)
Supplement: Supplementary file 3 [file pone.0356889.s003.docx]

Dear Editor,

I studied the revised manuscript, carefully. Regarding my previous comments, there is no any considerable change employed in the revised manuscript. For example, I suggested to the authors that there are two similar studies related to this manuscript. The authors should study these articles and compare their study with these articles.

Therefore, I repeat the previous recommendations to enhance the quality of this manuscript as follows.

This paper presents a novel method to improve the performance of AdaBoost in environments with noisy and imbalanced data. The paper claims novelty in improving AdaBoost; however, the authors should compare their proposed method with two similar studies listed below:

1. A noise-detection based AdaBoost algorithm for mislabeled data
2. Bounded exponential loss function based AdaBoost ensemble of OCSVMs

If the proposed method is similar to those presented in the two above-mentioned articles, the authors should clearly explain the similarities and differences.

In addition, the experimental results section should be improved. For example, the authors should describe the dataset in detail and explain the nature of the noise and class imbalance present in the data. To further improve the clarity of the paper, the authors are encouraged to review the two above-mentioned articles and follow their presentation structure. The results should be presented using figures and charts to enhance clarity. Furthermore, the quality of the figures should be improved.

PLOS One is a prestigious journal and the papers published in this journal should mind the basic and essential issues required to increase the credibility of them. The authors should consider the recommendations presented by the referees due to their valuable time that they consume to read and evaluate their manuscripts.

Regards.
