# Supplementary material for: ASWBoost: Classification algorithm for noisy and imbalanced data based on parametric exponential loss
Source: PLoS One. 2026 Aug 27;21(8):e0356889. doi: 10.1371/journal.pone.0356889 (PMC13521417; doi:10.1371/journal.pone.0356889)
Supplement: Supplementary file 4 [file pone.0356889.s004.docx]

**Response to Reviewers**

Title: *ASWBoost: A Robust Boosting Algorithm for Noisy and Imbalanced Classification via Parameterized Exponential Loss*

Manuscript ID: PONE-D-25-60888R1

Journal: PLOS ONE

Article Type: Research Article

Corresponding Author: Mei Yan

First Author: Fei Meng

Order of Authors: Fei Meng; Mei Yan; Hang Liu; Wan Xu; Hao Li

### *Message to the Academic Editor*

***Dear Academic Editor, Dr. Kaywan Othman Ahmed,***

*We would like to extend our most sincere gratitude to you for handling our manuscript, "ASWBoost: A Robust Boosting Algorithm for Noisy and Imbalanced Classification via Parameterized Exponential Loss" (PONE-D-25-60888R1). We deeply appreciate the efficient, professional, and rigorous editorial work you have dedicated to this submission.*

*Thank you for coordinating such an excellent panel of reviewers and for granting us the valuable opportunity to revise our paper. Guided by your decision letter and the reviewers' constructive feedback, our entire research team has made our utmost effort to fundamentally upgrade the manuscript. We have conducted a comprehensive structural overhaul, significantly expanded the theoretical and experimental analyses, and rigorously enhanced the statistical validations to ensure the highest scientific quality.*

*We are truly grateful for your time, patience, and unwavering commitment to maintaining the high standards of the journal. We sincerely hope that our comprehensive revisions successfully address all concerns and that the refined manuscript is now deemed acceptable for publication in PLOS ONE. We wish you great health, joy, and continued success in all your endeavors!*

*Sincerely,*

***The Authors***

**Dear Academic Editor and Reviewers,**

Thank you very much for your thorough evaluation of our manuscript and for providing such insightful and constructive feedback. We deeply appreciate the time and expertise you have dedicated to reviewing our work. Your comments have been invaluable in helping us significantly elevate the theoretical depth, structural clarity, and statistical rigor of our paper.

We have carefully considered each of the points raised and have extensively revised the manuscript accordingly. Below, we provide a detailed, point-by-point response to all comments from the Editor and the Reviewers. Corresponding modifications have been highlighted in the ‘Revised Manuscript with Track Changes’ document.

Best regards,

Mei Yan

**Response to Reviewer #1**

**Point 1.1:** *Funding: The Financial Disclosure field states, “The author(s) received no specific funding for this work” while the Funding section identifies Grant No. 202405AS350003. These statements are contradictory and must be reconciled before publication.*

**Point taken.**

Response:

We sincerely apologize for this administrative oversight during the initial online submission process. The information provided in the manuscript's “Funding” section is correct: this work was indeed supported by the Cross-integration Innovation Team of Modern Applied Mathematics and Life Sciences in Yunnan Province, China (Grant No. 202405AS350003).

To perfectly reconcile this contradiction and comply with PLOS ONE guidelines, we have taken the following corrective actions:

- We have updated the “Financial Disclosure” field in the Editorial Manager system during this resubmission to accurately reflect the aforementioned grant.
- We have explicitly included the corrected and updated Financial Disclosure statement in our Cover Letter.

**Point 1.2:** *Abstract: The statement “ASWBoost outperforms standard AdaBoost and other mainstream ensemble algorithms” overstates the findings. Authors should consider naming the specific ensemble algorithms over which ASWBoost demonstrates statistically significant superiority and qualify the claim accordingly.*

**Done.**

Response:

We completely agree with your assessment. Broad claims without specific context can inadvertently overstate the findings. We have revised the Abstract to explicitly name the exact algorithms that ASWBoost significantly outperforms based on our rigorous statistical tests, and we have objectively qualified its performance relative to XGBoost.

- Revision in Abstract: “Critically, statistical analyses confirm that ASWBoost significantly outperforms AdaBoost, CS-AdaBoost, GBDT, SMOTEBoost, and RUSBoost, with overall performance statistically comparable to XGBoost.”

We would like to express our most sincere gratitude for your continuous support and your meticulous review of our manuscript. We deeply appreciate your kind well wishes ("Good luck!") and your precise final suggestions regarding the funding disclosure and the abstract. Your exceptionally keen eye for detail has helped us correct the administrative oversight and perfect the final version of this paper. Thank you once again for your invaluable time, patience, and expertise throughout the entire review process. We wish you all the best in your future research and endeavors, along with great health and success!

**Response to Reviewer #2**

Dear Reviewer #2,

We would like to express our deepest gratitude for your meticulous review and your exceptional patience in evaluating our manuscript. We sincerely apologize that our previous revision did not fully grasp the depth of your highly valuable suggestions. We have taken your comments to heart, and over the past weeks, our entire research team has made our utmost effort to completely overhaul the manuscript. We have rigorously studied the literature you recommended, fundamentally re-architected our presentation structure, and thoroughly upgraded our figures. We hope you will find that these comprehensive modifications, which are explicitly detailed below and highlighted in the ‘Revised Manuscript with Track Changes’, thoroughly address your concerns and meet the rigorous standards of *PLOS ONE*.

**Point 2.1**: *This paper presents a novel method to improve the performance of AdaBoost in environments with noisy and imbalanced data. The paper claims novelty in improving AdaBoost; however, the authors should compare their proposed method with two similar studies listed below: 1. A noise-detection based AdaBoost algorithm for mislabeled data. 2. Bounded exponential loss function based AdaBoost ensemble of OCSVMs. If the proposed method is similar to those presented in the two above-mentioned articles, the authors should clearly explain the similarities and differences.*

**Response:** Thank you for directing us to these two foundational and state-of-the-art studies. We completely agree that a rigorous mathematical and conceptual comparison with these specific methods is essential to properly articulate the exact novelty of our ASWBoost algorithm. To thoroughly resolve this, we have incorporated your suggestions into two critical parts of our revised manuscript:

1. **In the Introduction (Section 1):** We have explicitly introduced these two studies to outline the current trajectory of robust boosting research. We acknowledged their significant contributions in mitigating AdaBoost's vulnerability to outliers and mislabeled data by preventing unbounded weight growth. This sets the stage for our motivation.
2. **In the newly added theoretical section (Section 3.5: Connection and Distinction with Existing Robust Boosting Frameworks):** We provided an in-depth, dedicated comparison detailing the exact similarities and differences:
   - **Differences from ND-AdaBoost (Cao et al., 2012):** ND-AdaBoost utilizes explicit noise-detection functions (e.g., via k-NN or EM algorithms) embedded into the loss function to assign noise labels iteratively. While highly effective, this explicit pre-filtering incurs substantial computational overhead. In contrast, ASWBoost achieves robust weight adjustment implicitly by parametrically reshaping the exponential loss via $\theta$, avoiding the need for external distance metrics and maintaining higher algorithmic efficiency.
   - **Differences from BELF-AEOCSVMs (Xing et al., 2024):** Xing et al. elegantly introduced a bounded exponential loss function to cap the penalty for outliers. However, as mathematically demonstrated in their study, their modified loss function is non-convex, which necessitates complex numerical methods (like the Newton-Raphson approach) for optimization. ASWBoost, conversely, strictly preserves the desirable convexity of the exponential loss function, thereby guaranteeing stable global convergence for standard multi-class imbalanced classification tasks.

**Point 2.2:** *In addition, the experimental results section should be improved. For example, the authors should describe the dataset in detail and explain the nature of the noise and class imbalance present in the data.*

**Response:** We deeply appreciate this insightful suggestion. We fully agree that explicitly clarifying the nature of the data is crucial for readers to understand why ASWBoost is practically valuable. We have substantially expanded **Section 4.4 (Performance on OpenML Datasets)** to detail these intrinsic characteristics:

- **Nature of Class Imbalance:** We explicitly explained that the severe class imbalance in the selected medical datasets (e.g., Mammography, Hypothyroid) stems from the natural epidemiological rarity of positive disease instances relative to healthy baselines.
- **Nature of Noise:** We clarified that the noise in these real-world datasets is dual-faceted: it comprises *feature noise* (e.g., instrumental artifacts or sensor inaccuracies) and *label noise* (e.g., driven by subjective diagnostic discrepancies among practitioners).
- We further linked these characteristics to our algorithm, explaining precisely how ASWBoost’s parametrically modified loss function dynamically caps the weight penalties on these overlapping, anomalous samples, preventing the model from memorizing noise.

**Point 2.3:** *To further improve the clarity of the paper, the authors are encouraged to review the two above-mentioned articles and follow their presentation structure.*

**Response:** Thank you for this highly professional guidance. We carefully reviewed the presentation paradigm of Cao et al. (2012) and Xing et al. (2024) and realized that our previous draft mixed background knowledge with theoretical derivations, which hindered clarity. To provide a top-tier reading experience, we have performed a massive structural overhaul of the entire manuscript:

1. **Added a “Preliminaries” Section (Section 2):** We isolated the standard AdaBoost algorithm (2.1) and the standard exponential loss definitions (2.2) to clearly separate existing knowledge from our novel contributions.
2. **Consolidated Methodology and Theory (Section 3):** We restructured the proposed ASWBoost into a highly logical flow: starting with the parametric loss function (3.1), deriving update formulae (3.2), proving the error bounds theoretically (3.3), explaining the algorithm's mechanism (3.4), and concluding with the aforementioned theoretical comparison to existing frameworks (3.5).
3. **Refined Experimental Layout (Section 4):** We reorganized the experiments into dedicated sub-sections (Synthetic data, Real-world data, Complexity, and Statistical Significance Analysis) to provide a systemic validation.

**Point 2.4:** *The results should be presented using figures and charts to enhance clarity. Furthermore, the quality of the figures should be improved.*

**Response:** We fully accept this criticism and have taken decisive actions to elevate the visual quality and clarity of our manuscript:

1. **Transforming Tables to Charts:** Following your advice to enhance visual clarity, we converted the previously tabular G-mean results into a highly intuitive bar chart (now **Fig 6**). This allows readers to directly and visually compare the performance trends across AUC, Balanced Accuracy, and G-mean simultaneously.
2. **Upgrading Figure Quality to Publication Standards:** We discarded all previous low-resolution plots. Our team utilized Python (matplotlib) to completely redraw every single figure (Figures 2, 3, 4, 5, 6, and 7). All figures have been exported in lossless **TIFF format with ultra-high resolution (300 to 600 DPI)**, ensuring crisp fonts, standardized layouts, and absolute clarity, fully meeting the stringent publication requirements of *PLOS ONE*.

We cannot overstate how much your rigorous and constructive feedback has improved the caliber of our research. We have poured our utmost dedication into this revision to ensure every single one of your concerns has been thoroughly and scientifically resolved. We sincerely hope that you find this heavily revised manuscript satisfactory and acceptable for publication.

### Response to Reviewer #3

**Dear Reviewer #3,**

We would like to express our deepest gratitude for your thorough, highly professional, and constructive review of our manuscript. We are particularly grateful for your encouraging remarks regarding the substantial improvements in our organization and methodology descriptions. Your meticulous attention to detail—ranging from figure labels and dataset characteristics to the rigorousness of our statistical analyses—has been instrumental in elevating our manuscript to a much higher scientific standard.

We have made our utmost effort to carefully address each of your insightful comments. Below, please find our detailed, point-by-point responses. The corresponding modifications have been highlighted in the *‘Revised Manuscript with Track Change’*.

**Point 3.1:** *In Figure 2, the label of the x-axis appears to be incorrect. Based on the context and the experimental setup, the x-axis should be related to the level of noise interference. The authors are encouraged to verify and correct the axis description.*

**Response:** Thank you for catching this error with such careful observation. You are completely correct. The x-axis in Figure 2 was inadvertently mislabeled during the drafting process. We have verified and corrected the x-axis label in the revised **Figure 2** to accurately read **“Noise Level (%)”** instead of the previous incorrect description.

**Point 3.2:** *For Table 2, it would be beneficial to provide additional information regarding the datasets, including the number of classes and the number of instances in each class. Such information would help readers better understand the characteristics and imbalance level of the datasets used in the experiments.*

**Response:** We sincerely appreciate this constructive suggestion. We fully agree that providing the exact instance distribution and the number of classes is crucial for readers to accurately comprehend the intrinsic complexity and the precise imbalance severity of the datasets used.

Accordingly, we have substantially expanded **Table 2** (in Section 4.4) in the revised manuscript. We added new columns to explicitly report the **“Original Classes”**, **“Majority Instances (Negative Class)”**, and **"Minority Instances (Positive Class)"**. Furthermore, we updated the introductory paragraph in **Section 4.4** to explain how multi-class datasets (e.g., Hypothyroid, Cardiotocography, and Dermatology) were converted into highly imbalanced binary classification tasks for this specific study, ensuring maximum clarity and transparency.

**Point 3.3:** *The presentation of the experimental results is currently inconsistent across evaluation metrics. Specifically, G-mean scores are reported in tabular form (Table 3), whereas AUC and Balanced Accuracy are presented only through bar charts. To improve consistency and facilitate a more precise comparison among methods, it is recommended that all evaluation metrics be reported in tabular form. This would allow readers to clearly examine the exact performance values achieved by each method.*

**Response:** We sincerely thank you for pointing out this inconsistency. We completely agree that a consistent presentation format across all evaluation metrics is absolutely essential for a high-quality paper, and we deeply respect your request to facilitate a precise numerical comparison among methods.

While we initially intended to convert all figures into tables as you recommended, another reviewer (Reviewer #2) explicitly requested the exact opposite: *“The results should be presented using figures and charts to enhance clarity”*.

To strike an optimal balance and perfectly reconcile both expert opinions, we have adopted the following comprehensive solution:

1. **Consistent Graphical Presentation:** We have standardized the format by converting the original G-mean table into a high-resolution bar chart (now presented as **Figure 6**). Thus, AUC, Balanced Accuracy, and G-mean are now consistently presented visually, satisfying Reviewer #2's request for intuitive global trend comparisons.
2. **Explicit Numerical Annotations:** To strictly fulfill your highly valid requirement that readers must be able to *“clearly examine the exact performance values”*, **we have explicitly annotated the exact numerical scores (e.g., 0.8647, 0.9562, etc.) directly above the bars for our proposed algorithm (ASWBoost) across all charts (Figures 4, 5, and 6).**

This strategy prevents the manuscript from becoming overly cluttered with massive tables, enhances the visual clarity of the trends, while strictly ensuring that the exact, precise performance values of our proposed method are transparently and rigorously reported for readers to examine. We hope you find this balanced approach satisfactory.

**Point 3.4:** *Since the study involves two different types of datasets, i.e. synthetic datasets and real-world datasets, it is recommended that the captions of all tables and figures explicitly indicate the dataset category to which the results correspond. This would help readers avoid potential confusion when interpreting the experimental findings.*

**Response:** This is a highly meticulous and excellent point. We completely agree that clearly distinguishing the dataset categories within the captions significantly enhances the self-explanatory nature of the figures and tables, thereby preventing any potential reader confusion.

To achieve the highest standard of clarity, we have conducted a "carpet-style" revision of all figure and table captions throughout the manuscript. Every single caption in Section 4 now explicitly specifies whether the presented results were derived from the **“synthetic dataset”** (e.g., Figures 2, 3, and Table 1) or the **“12 real-world OpenML datasets”** (e.g., Table 2, Figures 4, 5, 6, and 7). We believe these targeted revisions substantially improve the readability and structural rigor of our experimental section.

**Point 3.5:** *The experiments conducted on synthetic datasets include Macro-F1 as one of the evaluation metrics. However, this metric is not reported in the experiments on real-world datasets. The rationale behind this inconsistency should be clarified. If Macro-F1 is considered relevant for evaluating the synthetic datasets, it would be reasonable to include it for the real-world datasets as well, unless there is a specific justification for its omission.*

**Response:** We genuinely appreciate your rigorous observation regarding this metric inconsistency. Although Macro-F1 is informative for moderately imbalanced synthetic data, it was deliberately omitted in real-world evaluations because its inherent mathematical reliance on *Precision* renders it excessively volatile and potentially misleading under extreme class imbalance ratios (e.g., 42:1 in the Mammography dataset).

Consequently, to prevent misleading interpretations caused by extreme class priors in real-world medical data, we exclusively prioritized prevalence-independent metrics: AUC, Balanced Accuracy, and G-mean. These metrics provide a much more robust and objective assessment of a model's true discriminative capability in severe scenarios. Following your advice, we have explicitly incorporated this scientific rationale into **Section 4.4** of the revised manuscript to ensure complete transparency.

**Point 3.6 & 3.7:** *The statistical significance analysis appears to be conducted only for the G-mean metric. This practice is uncommon in the related literature, where statistical tests are generally performed for all reported evaluation metrics. The authors are encouraged to extend the statistical analysis to AUC, Balanced Accuracy, and any other reported metrics. The manuscript would further benefit from reporting the average ranks obtained from the Friedman test. Average ranks provide valuable insights into the relative performance differences among competing methods and are widely used to support the interpretation of statistical comparison results...*

**Response:** We sincerely thank you for this highly constructive methodological guidance. We completely agree that performing statistical tests exclusively on a single metric provides an incomplete picture, and that explicitly reporting average ranks is essential for scientific interpretability.

In the revised manuscript, we have comprehensively overhauled **Section 4.5 (Statistical Significance Analysis)** following the authoritative statistical guidelines for machine learning (Demšar, 2006, JMLR):

1. **Extended Statistical Analysis:** We have extended the non-parametric Friedman test and the Bonferroni-Dunn post-hoc test to encompass all primary evaluation metrics: AUC, Balanced Accuracy, and G-mean.
2. **Reporting Average Ranks:** We introduced a new table (**Table 4** in the revised text) that explicitly reports the exact average ranks of all 7 algorithms across these three metrics, greatly enhancing the interpretability of our statistical claims.
3. **Updated Visuals:** We provided new, standardized Critical Difference (CD) diagrams (**Fig 7**) for all three metrics to visually and mathematically support the interpretation of the statistical findings. This multidimensional statistical validation firmly substantiates the robust superiority of ASWBoost.

Once again, we extend our heartfelt gratitude for your exceptional patience and your rigorous, expert feedback. We have poured our utmost dedication into this revision to ensure every single one of your concerns has been thoroughly, scientifically, and elegantly resolved. We sincerely hope that our revised manuscript now meets your high expectations and the stringent standards of *PLOS ONE*.

### Response to Reviewer #5

**Dear Reviewer #5,**

We sincerely thank you for your highly positive evaluation and for officially recommending our manuscript for publication. We are incredibly grateful that you recognized our dedicated efforts in validating the proposed algorithm through comprehensive performance and statistical metrics.

Your constructive feedback during the review process has been absolutely instrumental in improving the scientific rigor and overall quality of our work. Thank you for generously sharing your time and expertise with us. We send you our warmest regards and wish you continued success and joy in all your professional and personal pursuits!

### Response to Reviewer #6

**Dear Reviewer #6,**

We would like to express our deepest appreciation for your incredibly encouraging comments. It is immensely rewarding for our research team to hear your conclusion that our paper presents a "genuine improvement" and is suitable for publication. We are particularly grateful for your positive recognition of our strengthened theoretical derivations, the substantially expanded experimental section, and the rigorous statistical validations.

Your high academic standards have greatly elevated the caliber of our manuscript. Thank you once again for your immense help and insightful guidance. We wish you all the very best, with abundant success and prosperity in the future!
