# Supplementary material for: ASWBoost: Classification algorithm for noisy and imbalanced data based on parametric exponential loss
Source: PLoS One. 2026 Aug 27;21(8):e0356889. doi: 10.1371/journal.pone.0356889 (PMC13521417; doi:10.1371/journal.pone.0356889)
Supplement: Peer review history

# Peer review: Decision Letter 0

**DOI:** 10.1371/journal.pone.0356889.r001

**Authors:** Kaywan Othman Ahmed

20 Jan 2026

Dear Dr. Yan,

Thank you for submitting your manuscript to PLOS ONE. After careful consideration, we feel that it has merit but does not fully meet PLOS ONE’s publication criteria as it currently stands. Therefore, we invite you to submit a revised version of the manuscript that addresses the points raised during the review process.

Please submit your revised manuscript by Mar 06 2026 11:59PM. If you will need more time than this to complete your revisions, please reply to this message or contact the journal office at plosone@plos.org. When you're ready to submit your revision, log on to https://www.editorialmanager.com/pone/ and select the 'Submissions Needing Revision' folder to locate your manuscript file.

- A letter that responds to each point raised by the academic editor and reviewer(s). You should upload this letter as a separate file labeled 'Response to Reviewers'.
- A marked-up copy of your manuscript that highlights changes made to the original version. You should upload this as a separate file labeled 'Revised Manuscript with Track Changes'.
- An unmarked version of your revised paper without tracked changes. You should upload this as a separate file labeled 'Manuscript'.

If you would like to make changes to your financial disclosure, please include your updated statement in your cover letter. Guidelines for resubmitting your figure files are available below the reviewer comments at the end of this letter.

If applicable, we recommend that you deposit your laboratory protocols in protocols.io to enhance the reproducibility of your results. Protocols.io assigns your protocol its own identifier (DOI) so that it can be cited independently in the future. For instructions see: https://journals.plos.org/plosone/s/submission-guidelines#loc-laboratory-protocols. Additionally, PLOS ONE offers an option for publishing peer-reviewed Lab Protocol articles, which describe protocols hosted on protocols.io. Read more information on sharing protocols at https://plos.org/protocols?utm_medium=editorial-email&utm_source=authorletters&utm_campaign=protocols.

We look forward to receiving your revised manuscript.

Kind regards,

Kaywan Othman Ahmed

Academic Editor

PLOS One

Journal Requirements:

When submitting your revision, we need you to address these additional requirements.

1. Please ensure that your manuscript meets PLOS ONE's style requirements, including those for file naming. The PLOS ONE style templates can be found at

https://journals.plos.org/plosone/s/file?id=wjVg/PLOSOne_formatting_sample_main_body.pdf and

https://journals.plos.org/plosone/s/file?id=ba62/PLOSOne_formatting_sample_title_authors_affiliations.pdf.

2. Please note that PLOS One has specific guidelines on code sharing for submissions in which author-generated code underpins the findings in the manuscript. In these cases, we expect all author-generated code to be made available without restrictions upon publication of the work. Please review our guidelines at https://journals.plos.org/plosone/s/materials-and-software-sharing#loc-sharing-code and ensure that your code is shared in a way that follows best practice and facilitates reproducibility and reuse.

3. We suggest you thoroughly copyedit your manuscript for language usage, spelling, and grammar. If you do not know anyone who can help you do this, you may wish to consider employing a professional scientific editing service.

The American Journal Experts (AJE) (https://www.aje.com/) is one such service that has extensive experience helping authors meet PLOS guidelines and can provide language editing, translation, manuscript formatting, and figure formatting to ensure your manuscript meets our submission guidelines. Please note that having the manuscript copyedited by AJE or any other editing services does not guarantee selection for peer review or acceptance for publication.

Upon resubmission, please provide the following:

The name of the colleague or the details of the professional service that edited your manuscript

A copy of your manuscript showing your changes by either highlighting them or using track changes (uploaded as a *supporting information* file)

A clean copy of the edited manuscript (uploaded as the new *manuscript* file)”.

4. We note that the grant information you provided in the ‘Funding Information’ and ‘Financial Disclosure’ sections do not match.

When you resubmit, please ensure that you provide the correct grant numbers for the awards you received for your study in the ‘Funding Information’ section.

5. We note that your Data Availability Statement is currently as follows: [All relevant data are within the manuscript and its Supporting Information files]

Please confirm at this time whether or not your submission contains all raw data required to replicate the results of your study. Authors must share the “minimal data set” for their submission. PLOS defines the minimal data set to consist of the data required to replicate all study findings reported in the article, as well as related metadata and methods (https://journals.plos.org/plosone/s/data-availability#loc-minimal-data-set-definition).

For example, authors should submit the following data:

- The values behind the means, standard deviations and other measures reported;

- The values used to build graphs;

- The points extracted from images for analysis.

Authors do not need to submit their entire data set if only a portion of the data was used in the reported study.

If your submission does not contain these data, please either upload them as Supporting Information files or deposit them to a stable, public repository and provide us with the relevant URLs, DOIs, or accession numbers. For a list of recommended repositories, please see https://journals.plos.org/plosone/s/recommended-repositories.

If there are ethical or legal restrictions on sharing a de-identified data set, please explain them in detail (e.g., data contain potentially sensitive information, data are owned by a third-party organization, etc.) and who has imposed them (e.g., an ethics committee). Please also provide contact information for a data access committee, ethics committee, or other institutional body to which data requests may be sent. If data are owned by a third party, please indicate how others may request data access.

5. We note you have included a table to which you do not refer in the text of your manuscript. Please ensure that you refer to Table 4 & 5 in your text; if accepted, production will need this reference to link the reader to the Table

6. If the reviewer comments include a recommendation to cite specific previously published works, please review and evaluate these publications to determine whether they are relevant and should be cited. There is no requirement to cite these works unless the editor has indicated otherwise.

[Note: HTML markup is below. Please do not edit.]

Reviewers' comments:

Reviewer's Responses to Questions

**Comments to the Author**

1. Is the manuscript technically sound, and do the data support the conclusions?

Reviewer #1: Yes

Reviewer #2: Yes

Reviewer #3: Yes

Reviewer #4: Yes

Reviewer #5: Yes

**********

2. Has the statistical analysis been performed appropriately and rigorously?

Reviewer #1: Yes

Reviewer #2: Yes

Reviewer #3: No

Reviewer #4: No

Reviewer #5: N/A

**********

3. Have the authors made all data underlying the findings in their manuscript fully available?

The PLOS Data policy

Reviewer #1: No

Reviewer #2: Yes

Reviewer #3: Yes

Reviewer #4: No

Reviewer #5: Yes

**********

4. Is the manuscript presented in an intelligible fashion and written in standard English?

Reviewer #1: No

Reviewer #2: Yes

Reviewer #3: Yes

Reviewer #4: No

Reviewer #5: Yes

**********

Reviewer #1: Review Comments to the Author for Consideration

The manuscript reflects a strong level of technical depth; however, several aspects of presentation, structure, and methodological clarity would benefit from improvement to enhance readability, transparency, and overall academic quality.

The manuscript would benefit from a more explicitly defined problem statement. While the need for the research can be inferred, the authors should clearly articulate the problem being addressed, including not only the algorithmic improvement but also the rationale for its application to the selected dataset and practical use case.

The opening sentence of the introduction, “When strong learners are difficult to learn directly in machine learning, Boosting provides an effective design framework for ensemble learning,” should be revised. As the first sentence of an academic manuscript, it should reflect a more polished scholarly tone, avoid awkward phrasing, and clearly establish context with sufficient technical depth. In addition, the introductory paragraph would benefit from improved logical flow and cohesion, as statements such as “AdaBoost has good generalization ability and is not prone to overfitting” appear somewhat isolated without clearer linkage to preceding sentences.

The repeated use of the phrase “In practical applications” throughout the introduction is stylistically repetitive and should be reviewed. Similarly, the frequent use of “the AdaBoost” is, in many instances, grammatically unnecessary and overly repetitive and should be corrected to align with standard academic usage.

In the sentence “This mechanism forces subsequent weak classifiers to overly focus on noisy samples, thereby seriously reducing the learning performance of the model,” the expressions “overly focus” and “seriously reducing” appear subjective and insufficiently objective for academic writing. More measured and precise phrasing is recommended. In addition, multiple instances of “[Error! Reference source not found.]” should be corrected.

The paragraph outlining the paper structure (e.g., “The structure of this paper is as follows…”) does not appear necessary in the introduction of an academic research article and should be reviewed, particularly if not required by the journal’s formatting guidelines.

The authors should consider reorganising the overall manuscript structure and adopting more professional and standardised chapter or section headings. In particular, clearer separation between the background, the proposed methodology, theoretical analysis, experimental evaluation, results, and conclusion would significantly improve readability and accessibility for a broad audience.

From a methodological perspective, the authors should clarify whether alternative feature selection algorithms were considered and provide a brief justification for the selection of the Boruta algorithm, including the criteria or comparative considerations that informed this choice.

The authors should also consider including the URL link to the Statlog dataset utilised and provide an overview of the dataset background, including a clear rationale for its selection in the context of the study.

The presentation of the evaluation results would benefit from improvement. Specifically, the authors should consider utilising a consistent table orientation aligned with the main text, as the current presentation disrupts readability and makes comparison of results less intuitive.

In addition, figures directly supporting the reported results should be presented within the relevant results or discussion sections, rather than being indexed at the later end of the manuscript, as this would improve interpretability and allow readers to more easily relate visual evidence to the accompanying analysis.

The concluding recommendations "Furthermore, based on the research findings, recommendations for the prevention of cardiovascular diseases ... early intervention and reducing the risk of disease onset" should be strengthened by explicitly anchoring them to the model outputs and feature-level evidence derived from the analysis.

Finally, the authors should note that the abstract should also reflect these improvements, such that it effectively conveys its intended purpose of providing a comprehensive and coherent overview of the study.

Overall, the study reflects strong technical depth but would significantly benefit from the improvements outlined above.

Good work to the authors, and good luck!

Reviewer #2: This paper presents a novel method to improve the performance of AdaBoost in environments with noisy and imbalanced data. The paper claims novelty in improving AdaBoost; however, the authors should compare their proposed method with two similar studies listed below:

1. A noise-detection based AdaBoost algorithm for mislabeled data

2. Bounded exponential loss function based AdaBoost ensemble of OCSVMs

If the proposed method is similar to those presented in the two above-mentioned articles, the authors should clearly explain the similarities and differences.

In addition, the experimental results section should be improved. For example, the authors should describe the dataset in detail and explain the nature of the noise and class imbalance present in the data. To further improve the clarity of the paper, the authors are encouraged to review the two above-mentioned articles and follow their presentation structure. The results should be presented using figures and charts to enhance clarity. Furthermore, the quality of the figures should be improved.

Reviewer #3: This paper proposes a Smooth Bounded Exponential Loss–based AdaBoost method for classification on noisy and imbalanced datasets. The proposed method extends the standard AdaBoost algorithm by introducing a parameter, “theta,” into the classifier weight calculation. While the idea is potentially interesting, the current manuscript requires substantial revisions before it can be considered for publication. In particular, the paper lacks a clear and well-structured explanation of the theoretical motivation behind the proposed loss function and weighting mechanism. Moreover, the experimental evaluation and result analysis are not sufficiently comprehensive to fully support the claimed improvements over existing methods. Addressing these issues would significantly improve the clarity, rigor, and overall quality of the paper. The following points detail my major concerns:

1. The proposed ASWBoost incorporates the parameter “theta” into the classifier weight calculation, as presented in Equations (2), (4), and (5). The authors have provided the general motivation and mechanism of using theta to control classifier weights. However, it would be beneficial to include a more intuitive explanation of how theta in Equations (2), (4), and (5) affects the smoothness of both classifier weights and sample weights. Such an explanation would help readers better understand the technical role of theta and how it influences the learning process.

2. The authors have presented all the required equations for ASWBoost in the manuscript. Therefore, I suggest simplifying the notation in Algorithm 1 by removing the explicit equations and instead referring to the corresponding equations introduced earlier in the paper. Repeating the same equations within the algorithm is unnecessary and may reduce clarity. In addition, it is unclear whether Equations (8) and (9) are part of the ASWBoost procedure described in Algorithm 1. If they are, Algorithm 1 should be revised accordingly to explicitly include these equations.

3. The proposed method has been evaluated extensively using synthetic datasets with varying sample sizes and noise levels. However, the study lacks evaluation on real-world datasets, particularly publicly available benchmark datasets commonly used in class imbalance classification research. Real-world datasets often exhibit more complex characteristics, such as irregular data distributions, uncorrelated or redundant features, high dimensionality, severe class overlap, and the presence of small disjuncts. In my view, evaluating the proposed method solely on synthetic datasets is not sufficient when many public benchmark datasets are readily available. Therefore, I strongly recommend that the authors consider evaluating their method on real-world benchmark datasets and refer to the following relevant studies:

a. Azal Ahmad Khan, Omkar Chaudhari, Rohitash Chandra, “A review of ensemble learning and data augmentation models for class imbalanced problems: Combination, implementation and evaluation,” Expert Systems with Applications, Volume 244, 2024. https://doi.org/10.1016/j.eswa.2023.122778.

b. Fachrie, M., Musdholifah, A., and Pulungan, R., “Effectiveness of data resampling and ensemble learning in multiclass imbalance learning,” Artificial Intelligence Review, Volume 58, 368 (2025). https://doi.org/10.1007/s10462-025-11357-w

4. Since the paper focuses on handling class imbalance, I recommend evaluating the classification performance of ASWBoost using metrics that are more appropriate for imbalanced learning, such as Average Accuracy (AvAcc), Geometric Mean (G-mean), macro F1-score, and AUC. Metrics such as accuracy, precision, recall, and standard F1-score are often misleading in imbalanced classification settings. Please also provide clear and correct mathematical definitions for all evaluation metrics used in this study.

5. As theta is the key parameter that plays a significant role in ASWBoost, it is necessary to provide an in-depth analysis of how different values of theta influence classification performance. I suggest conducting additional experiments using a range of theta values and presenting a comprehensive analysis of the relationship between theta and the performance of ASWBoost.

6. Please include appropriate statistical significance analysis, for example, using the Friedman test followed by a Nemenyi post-hoc test, to assess whether the performance differences between ASWBoost and other boosting algorithms are statistically significant.

7. Several entries in Tables 2, 3, and 4 are confusing, particularly the terms “200 datasets,” “3000 datasets,” and “10000 datasets.” It is unclear how experiments could be conducted using such a large number of datasets. I assume that the authors actually mean “200 instances,” “3000 instances,” and “10000 instances.” Please revise these tables to provide clear and accurate information.

8. Several citations in the manuscript are not properly recognized or formatted. Please carefully review and correct all references to ensure accuracy and consistency.

9. Finally, the conclusion should clearly discuss the limitations of the proposed method. As no method is without limitations, this discussion is essential for providing a balanced perspective. In addition, please outline potential directions for future work that could help other researchers extend or improve upon the current study.

Reviewer #4: The manuscript presents a modified AdaBoost variant (ASWBoost) using a smooth bounded exponential loss to improve robustness against noise and class imbalance. The topic is relevant and suitable for high impact factor journal, and the idea of controlling sample weight updates with a tunable parameter is meaningful. However, the paper currently suffers from mathematical inconsistencies, unclear derivations, missing experimental details, depth discuss of the experimental result, formatting errors, and insufficient comparison rigor, which must be addressed before the work can be considered for publication.

Reviewer #5: The paper discussed the enhanced version of AdaBoost that efficiently leverages imbalanced and noisy data. The author utilized real-world and benchmark datasets to prove their algorithm’s performance, which outperforms AdaBoost.

**********

what does this mean?). If published, this will include your full peer review and any attached files.

If you choose “no”, your identity will remain anonymous but your review may still be made public.

**Do you want your identity to be public for this peer review?**  For information about this choice, including consent withdrawal, please see our Privacy Policy

Reviewer #1: **Yes:** Adefemi Lawrence Ayodele

Reviewer #2: **Yes:** Mohammad Khanbabaei

Reviewer #3: No

Reviewer #4: No

Reviewer #5: No

**********

[NOTE: If reviewer comments were submitted as an attachment file, they will be attached to this email and accessible via the submission site. Please log into your account, locate the manuscript record, and check for the action link "View Attachments". If this link does not appear, there are no attachment files.]

To ensure your figures meet our technical requirements, please review our figure guidelines: https://journals.plos.org/plosone/s/figures

You may also use PLOS’s free figure tool, NAAS, to help you prepare publication quality figures: https://journals.plos.org/plosone/s/figures#loc-tools-for-figure-preparation.

NAAS will assess whether your figures meet our technical requirements by comparing each figure against our figure specifications.

Attachment Submitted filename: Reviewer Report-PONE-D-25-60888.docx

# Author comment: Author response to Decision Letter 1

**DOI:** 10.1371/journal.pone.0356889.r002

14 Apr 2026

Responses to comments from the AE

It was very considerate and kind of you to proofread our paper. Thank you very much for your critical and constructive comments. We have done our best to accommodate your comments and suggestions when revising our paper. For your reference, your comments are reproduced below, followed by our response.

Title Revision: We have revised the title to “ASWBoost: Classification Algorithm for Noisy and Imbalanced Data Based on Parametric Exponential Loss.” We have removed the “Smooth Bounded” to avoid potential confusion. This term was originally intended to refer to the training error upper bound rather than the loss function itself. We apologize for this oversight and believe the new title more accurately reflects the scope of our work.

We have carefully studied all the comments and have made substantial revisions to the manuscript accordingly, including:

Novelty and Positioning Relative to Existing Work

The proposed method represents an incremental modification of AdaBoost through the introduction of a control parameter that moderates sample weight updates. Similar concepts have already been explored in the literature, including Bounded exponential loss function based AdaBoost ensemble of OCSVMs, cost-sensitive AdaBoost, AWABoost, RobustBoost, and self-paced boosting methods.

The manuscript does not clearly articulate how ASWBoost fundamentally differs from these existing approaches.

The contribution would be strengthened by a systematic comparison of mechanisms, explicitly highlighting what is new in the loss function design, optimization strategy, or theoretical guarantees.

Recommendation:

Include a comparative discussion or table summarizing differences between ASWBoost and closely related boosting variants, focusing on loss functions, parameter roles, noise handling, and imbalance handling.

Done.

Response: We sincerely thank the reviewer for this insightful comment. We agree that clarifying the theoretical positioning of ASWBoost relative to existing variants is crucial for demonstrating its contribution. We have carefully revised the manuscript to explicitly distinguish ASWBoost from RobustBoost, Cost-Sensitive Boosting, SPLBoost, and AWABoost.

Although ASWBoost introduces a parameter to control weight updates, this is not merely a heuristic modification. The parameter is derived from a modified exponential loss function that fundamentally alters the weight update strategy while preserving convexity.

In the revised Introduction sections, we have added a systematic comparison discussing the mechanisms, advantages, and limitations of these methods. The key distinctions are summarized as follows:

vs. RobustBoost (Non-convex Loss): RobustBoost employs a non-convex loss function based on Brownian motion, which destroys the convex optimization property of standard Boosting, leading to instability and sensitivity to hyperparameters. ASWBoost, in contrast, introduces a parameter into the exponential loss without truncation, preserving the convexity of the objective function. This ensures a stable global optimum and consistent convergence (as proven in our new Theorem 2).

vs. Cost-Sensitive AdaBoost (Fixed Costs): Cost-sensitive methods rely on a pre-defined, fixed cost matrix. This often leads to overfitting outliers in the minority class. ASWBoost does not use a fixed matrix; instead, the parameter dynamically adjusts the gradient of the loss function. This allows the model to focus on hard samples without blindly increasing the weights of potential noise, offering better robustness.

vs. SPLBoost (Explicit Self-Paced Learning): SPLBoost combines a classification loss with a self-paced regularization term to explicitly select easy samples, which is computationally complex. ASWBoost achieves a similar noise-filtering effect implicitly through its continuous weight adjustment mechanism. It down-weights noise naturally via the loss function curvature rather than requiring a hard/easy sample selection step, making it more computationally efficient.

vs. AWABoost (Heuristic Adjustment): AWABoost modifies the weight update step heuristically by inserting a control function to limit weight growth. Its robustness comes from constraining the procedure. In contrast, ASWBoost’s weight update rule is mathematically derived directly from minimizing our proposed objective function. This ensures theoretical consistency between the loss function and the algorithm’s behavior, guaranteeing the training error bound.

We appreciate the reviewer’s suggestion regarding the OCSVM-based AdaBoost ensemble. While we acknowledge its relevance to robust learning, we did not include it in our comparison for two primary reasons. First, to ensure a fair evaluation of the boosting framework itself, we maintained consistency by using decision stumps as the base learner for all methods. Introducing an SVM-based ensemble would make it difficult to discern whether performance variations arise from the loss function or the inherent difference in inductive bias between trees and SVMs. Second, OCSVM-based methods are typically tailored for one-class or anomaly detection tasks, whereas ASWBoost is designed for supervised binary classification under noise and imbalance. We appreciate the pointer and will consider exploring OCSVM-based ensembles in our future research.

Mathematical Consistency and Theoretical Soundness

Several mathematical issues require careful correction:

αₘ is defined multiple times with conflicting formulas, e.g.:

Equation (2):

α_m= 1/2 log⁡〖(1-e_m)/(e_m-θ)〗

Equation (16):

α_m^*= 1/(2(1+ θ)) log⁡〖(1-e_m)/e_m 〗

These are not equivalent, yet the paper claims they are identical.

The proposed “bounded exponential loss” is not rigorously shown to be bounded. Explicit upper and lower bounds are not provided.

Proofs of Theorems 2 and 3 are excessively long, difficult to follow, and contain notational inconsistencies and redundant steps.

Recommendation:

Use a single, consistent definition of αₘ and clearly derive it from loss minimization.

Explicitly prove or demonstrate the boundedness and smoothness of the proposed loss function.

Move lengthy derivations to an appendix and provide clearer, more concise proofs in the main text.

Done.

Response: Thank you for this important observation. We have re-examined the mathematical derivations and thoroughly revised Section 3 to ensure consistency and rigor. In particular, by minimizing the objective function (Eq. 11) using Fermat’s principle, we derive the optimal coefficient . As presented in the revised Section 3.1 (Eq. 15) and Algorithm 2, is given uniformly by

We agree with the reviewer’s assessment that the term ‘Bounded’ was imprecise, as the loss function is convex and smooth but not strictly bounded in a mathematical sense. Consequently, we have revised the manuscript title to “ASWBoost: Classification Algorithm for Noisy and Imbalanced Data Based on Parametric Exponential Loss.” Furthermore, we have standardized the mathematical notation throughout the paper, provided a rigorous proof for the training error bounds, and streamlined the derivation steps for improved clarity. As shown in Figure 1, the smoothness of our proposed loss function is now clearly illustrated.

3. Algorithm Description and Clarity

Although the ASWBoost algorithm is described in detail, the presentation lacks clarity:

The algorithm description repeats equations already given earlier without adding clarity.

Algorithm 1 repeats equations already stated earlier.

Notation switches between:

G(x),f(x)

ω_i,D_m

The role of θ > 0 vs θ < 0 is not algorithmically explicit

The practical interpretation of the parameter θ (positive vs. negative values) is not sufficiently illustrated.

Recommendation:

Provide a simplified and self-contained pseudocode.

Include a schematic or flowchart illustrating how θ affects weight updates in noisy versus imbalanced settings.

Done.

Response: To address these issues, we have comprehensively revised Section 3 and Algorithm 2. The specific improvements are as follows:

Simplified Pseudocode: As recommended, We retained Algorithm 1 to outline standard AdaBoost for baseline context, and we have provided a simplified, self-contained pseudocode for our proposed method in Algorithm 2.

Unified Notation: We have thoroughly proofread the manuscript and standardized all mathematical notations. We now consistently use to denote the base classifier and to represent the weight distribution, eliminating the confusion between mixed notations like or .

Visual Illustration of : To clarify the practical interpretation of the parameter , we have added a new schematic diagram, Figure 1 (Section 3.3), and expanded the corresponding discussion.

We explicitly explain that when , the algorithm imposes a larger penalty on misclassified samples, thereby increasing the focus on the minority class (Imbalance Handling).

Conversely, when , the weight update becomes more conservative to suppress the influence of outliers, enhancing robustness against label noise (Noise Handling).

4. Experimental Design and Evaluation

4.1 Synthetic Dataset Construction

The manuscript lacks sufficient detail regarding synthetic data generation:

Gaussian distribution parameters, noise injection mechanisms, and redundant feature construction are not fully specified.

This omission hinders reproducibility.

4.2 Baseline Comparisons

The experimental comparison is limited primarily to AdaBoost.

Important baselines such as Bounded exponential loss function based AdaBoost ensemble of OCSVMs, RobustBoost, cost-sensitive AdaBoost, Gradient Boosting, and modern ensemble methods (e.g., XGBoost) are missing.

No statistical significance tests are conducted.

Recommendation:

Include additional competitive baselines.

Apply statistical tests (e.g., Friedman test with post-hoc analysis) to support claims of superiority.

Done.

Response: In response to this comment, we have completely rewritten the experimental section (Section 4). Our specific revisions are as follows:

1. Regarding Synthetic Dataset Construction (Section 4.2) We have provided a detailed description of the synthetic data generation process to ensure reproducibility. Specifically:

Data Structure:we have utilized the make_classification tool to generate a synthetic dataset consisting of 1,000 samples and 40 features.

Feature Generation: We specified that 25 informative features are generated using Gaussian clusters with a standard deviation of 1.0 and a class separation factor of 1.5. Additionally, 10 redundant features are linear combinations of the informative ones, and 5 noise features are drawn from a standard normal distribution N(0,1).

Noise Injection: We explicitly stated that label noise was simulated by randomly flipping class labels at rates of 10%, 20%, and 30%.

2. Regarding Baseline Comparisons (Section 4.2 & 4.4) We have significantly expanded our comparative analysis. Instead of limiting the comparison to AdaBoost, we have introduced a comprehensive set of state-of-the-art and classic baselines:

Robustness Baselines: We added RobustBoost and Cost-Sensitive AdaBoost (CS-AdaBoost) to evaluate performance under noise and imbalance.

Advanced Ensemble Methods: We included GBDT and XGBoost as strong baselines to test the competitiveness of ASWBoost against modern gradient boosting frameworks.

Imbalance Handling Baselines: For the real-world datasets, we also compared against SMOTEBoost (oversampling) and RUSBoost (undersampling) to validate our re-weighting strategy.

3. Regarding Statistical Significance Tests (Section 4.4) To ensure the reliability of our conclusions, we have incorporated rigorous statistical testing. We employed the Friedman test to detect statistical differences among the algorithms across the 12 OpenML datasets. Upon rejecting the null hypothesis ( ), we conducted the Nemenyi post-hoc test to identify specific pairwise differences. The critical difference (CD) diagrams have been added as Figure 6 to visually present these results.

5. Real-World Dataset and Ethical Considerations

The cardiovascular disease dataset is used as a practical application, but critical information is missing:

Dataset source and preprocessing steps are not described.

The validation strategy (train/test split or cross-validation) is unclear.

No ethical approval or public-dataset statement is provided, despite involving medical data.

Recommendation:

Clearly state the dataset origin, ethical considerations, and validation methodology in compliance with PLOS ONE guidelines.

Done.

Response: Thank you for pointing out these critical omissions. We fully agree that transparency regarding data sources, preprocessing, and ethical compliance is essential. we have made the following substantial revisions in Section 4.4 (OpenML Datasets) and Table 2 of the revised manuscript:

Dataset Source and Expansion: We have replaced the single cardiovascular dataset with 12 publicly available benchmark datasets from the OpenML repository (https://www.openml.org/), which includes multiple medical datasets (e.g., Heart Failure, Mammography, Diabetes). Detailed statistics for these datasets are now listed in Table 2.

Preprocessing Details: We have explicitly described the data preprocessing steps. As stated in Section 4.4, all features were standardized to have a mean of 0 and a standard deviation of 1 to ensure consistent model training.

Validation Strategy: We have clarified our validation methodology. To ensure robust performance evaluation, we employed a stratified 5-fold cross-validation strategy for all experiments, as detailed in Section 4.1 and Section 4.4.

Ethical Considerations: We have included a statement regarding ethical compliance. Since this study exclusively utilizes publicly available, anonymized datasets from the OpenML repository, no specific ethical approval or patient consent was required.

6. Reproducibility and Transparency

The manuscript does not meet reproducibility expectations:

No code or implementation details are provided.

Random seed control and hyperparameter ranges are not specified.

Computational complexity and runtime comparisons are absent.

Recommendation:

Provide code availability, implementation details, and computational analysis to ensure reproducibility.

Done.

Response: We have addressed this comment by making the following substantial revisions:

1. Open Source Code: We have uploaded the complete source code, including the implementation of the ASWBoost algorithm and the scripts for reproducing the experiments, to a public GitHub repository.

Repository Link: https://github.com/064213mf/ASWBoost.git

This link has also been added to the code availability

2. Implementation Details & Random Seed: To ensure result reproducibility, we have explicitly detailed the experimental setup in Section 4 (Experimental Design and Evaluation):

Platform: All algorithms were implemented using Python 3.8 and the Scikit-learn library.

Random Seed: We standardized the random seed to 42 for all data splitting, model initialization, and noise generation processes to guarantee that all results are deterministic and reproducible.

Validation Strategy: We employed a 5-fold stratified cross-validation for the outer loop to evaluate performance, ensuring class distribution consistency across folds.

3. Hyperparameter Specifications: We have clarified the parameter settings in Section 4.4:

Proposed Method (ASWBoost): The smoothing parameter was tuned via grid search within the range of . The number of boosting iterations was set to 50.

Baselines: All comparison models (AdaBoost, Gradient Boosting, XGBoost, etc.) were constructed using Scikit-learn or their respective standard libraries with default parameters unless otherwise specified, with the iteration count uniformly set to 50 for fair comparison.

Complexity and Runtime Analysis: We have added a new subsection, Section 4.3 Complexity Analysis, to the revised manuscript.

Theoretical Analysis: We demonstrated that ASWBoost maintains the same computational complexity order as standard AdaBoost, which is , where N is the sample size and d is the feature dimension.

Empirical Compariso

Attachment Submitted filename: Response to Reviewers.docx

# Peer review: Decision Letter 1

**DOI:** 10.1371/journal.pone.0356889.r003

**Authors:** Kaywan Othman Ahmed

22 Jun 2026

Dear Dr. Yan,

Thank you for submitting your manuscript to PLOS ONE. After careful consideration, we feel that it has merit but does not fully meet PLOS ONE’s publication criteria as it currently stands. Therefore, we invite you to submit a revised version of the manuscript that addresses the points raised during the review process.

Please submit your revised manuscript by Aug 06 2026 11:59PM. If you will need more time than this to complete your revisions, please reply to this message or contact the journal office at plosone@plos.org. When you're ready to submit your revision, log on to https://www.editorialmanager.com/pone/ and select the 'Submissions Needing Revision' folder to locate your manuscript file.

- A letter that responds to each point raised by the academic editor and reviewer(s). You should upload this letter as a separate file labeled 'Response to Reviewers'.
- A marked-up copy of your manuscript that highlights changes made to the original version. You should upload this as a separate file labeled 'Revised Manuscript with Track Changes'.
- An unmarked version of your revised paper without tracked changes. You should upload this as a separate file labeled 'Manuscript'.

If you would like to make changes to your financial disclosure, please include your updated statement in your cover letter. Guidelines for resubmitting your figure files are available below the reviewer comments at the end of this letter.

If applicable, we recommend that you deposit your laboratory protocols in protocols.io to enhance the reproducibility of your results. Protocols.io assigns your protocol its own identifier (DOI) so that it can be cited independently in the future. For instructions see: https://journals.plos.org/plosone/s/submission-guidelines#loc-laboratory-protocols. Additionally, PLOS ONE offers an option for publishing peer-reviewed Lab Protocol articles, which describe protocols hosted on protocols.io. Read more information on sharing protocols at https://plos.org/protocols?utm_medium=editorial-email&utm_source=authorletters&utm_campaign=protocols.

As the corresponding author, your ORCID iD is verified in the submission system and will appear in the published article. PLOS supports the use of ORCID, and we encourage all coauthors to register for an ORCID iD and use it as well. Please encourage your coauthors to verify their ORCID iD within the submission system before final acceptance, as unverified ORCID iDs will not appear in the published article. *Only*  the individual author can complete the verification step; PLOS staff *cannot*  verify ORCID iDs on behalf of authors.

We look forward to receiving your revised manuscript.

Kind regards,

Kaywan Othman Ahmed

Academic Editor

PLOS One

Journal Requirements:

If the reviewer comments include a recommendation to cite specific previously published works, please review and evaluate these publications to determine whether they are relevant and should be cited. There is no requirement to cite these works unless the editor has indicated otherwise.

[Note: HTML markup is below. Please do not edit.]

Reviewers' comments:

Reviewer's Responses to Questions

**Comments to the Author**

Reviewer #1: All comments have been addressed

Reviewer #2: (No Response)

Reviewer #3: (No Response)

Reviewer #5: All comments have been addressed

Reviewer #6: All comments have been addressed

**********

2. Is the manuscript technically sound, and do the data support the conclusions?

Reviewer #1: Yes

Reviewer #2: Partly

Reviewer #3: Yes

Reviewer #5: Yes

Reviewer #6: Yes

**********

3. Has the statistical analysis been performed appropriately and rigorously?

Reviewer #1: Yes

Reviewer #2: N/A

Reviewer #3: No

Reviewer #5: I Don't Know

Reviewer #6: Yes

**********

4. Have the authors made all data underlying the findings in their manuscript fully available?

The PLOS Data policy

Reviewer #1: Yes

Reviewer #2: Yes

Reviewer #3: Yes

Reviewer #5: Yes

Reviewer #6: Yes

**********

5. Is the manuscript presented in an intelligible fashion and written in standard English?

Reviewer #1: Yes

Reviewer #2: Yes

Reviewer #3: Yes

Reviewer #5: Yes

Reviewer #6: Yes

**********

Reviewer #1: Thank you for submitting your revised manuscript.

However, the two minor revisions highlighted below require attention.

Funding: The Financial Disclosure field states, "The author(s) received no specific funding for this work" while the Funding section identifies Grant No. 202405AS350003. These statements are contradictory and must be reconciled before publication.

Abstract: The statement "ASWBoost outperforms standard AdaBoost and other mainstream ensemble algorithms" overstates the findings. Authors should consider naming the specific ensemble algorithms over which ASWBoost demonstrates statistically significant superiority and qualify the claim accordingly.

Thank you.

Good luck!

Reviewer #2: Dear Editor,

I studied the revised manuscript, carefully. Regarding my previous comments, there is no any considerable change employed in the revised manuscript. For example, I suggested to the authors that there are two similar studies related to this manuscript. The authors should study these articles and compare their study with these articles.

Therefore, I repeat the previous recommendations to enhance the quality of this manuscript as follows.

This paper presents a novel method to improve the performance of AdaBoost in environments with noisy and imbalanced data. The paper claims novelty in improving AdaBoost; however, the authors should compare their proposed method with two similar studies listed below:

1. A noise-detection based AdaBoost algorithm for mislabeled data

2. Bounded exponential loss function based AdaBoost ensemble of OCSVMs

If the proposed method is similar to those presented in the two above-mentioned articles, the authors should clearly explain the similarities and differences.

In addition, the experimental results section should be improved. For example, the authors should describe the dataset in detail and explain the nature of the noise and class imbalance present in the data. To further improve the clarity of the paper, the authors are encouraged to review the two above-mentioned articles and follow their presentation structure. The results should be presented using figures and charts to enhance clarity. Furthermore, the quality of the figures should be improved.

PLOS One is a prestigious journal and the papers published in this journal should mind the basic and essential issues required to increase the credibility of them. The authors should consider the recommendations presented by the referees due to their valuable time that they consume to read and evaluate their manuscripts.

Regards.

Reviewer #3: The manuscript has been substantially revised, and the current version shows a noticeable improvement in terms of organization, methodology description, and overall presentation. The authors have addressed many of the concerns raised in the previous review round. Nevertheless, several issues remain that should be considered to further improve the clarity, consistency, and completeness of the manuscript.

1. In Figure 2, the label of the x-axis appears to be incorrect. Based on the context and the experimental setup, the x-axis should be related to the level of noise interference. The authors are encouraged to verify and correct the axis description.

2. For Table 2, it would be beneficial to provide additional information regarding the datasets, including the number of classes and the number of instances in each class. Such information would help readers better understand the characteristics and imbalance level of the datasets used in the experiments.

3. The presentation of the experimental results is currently inconsistent across evaluation metrics. Specifically, G-mean scores are reported in tabular form (Table 3), whereas AUC and Balanced Accuracy are presented only through bar charts. To improve consistency and facilitate a more precise comparison among methods, it is recommended that all evaluation metrics be reported in tabular form. This would allow readers to clearly examine the exact performance values achieved by each method.

4. Since the study involves two different types of datasets, i.e. synthetic datasets and real-world datasets, it is recommended that the captions of all tables and figures explicitly indicate the dataset category to which the results correspond. This would help readers avoid potential confusion when interpreting the experimental findings.

5. The experiments conducted on synthetic datasets include Macro-F1 as one of the evaluation metrics. However, this metric is not reported in the experiments on real-world datasets. The rationale behind this inconsistency should be clarified. If Macro-F1 is considered relevant for evaluating the synthetic datasets, it would be reasonable to include it for the real-world datasets as well, unless there is a specific justification for its omission.

6. The statistical significance analysis appears to be conducted only for the G-mean metric. This practice is uncommon in the related literature, where statistical tests are generally performed for all reported evaluation metrics. The authors are encouraged to extend the statistical analysis to AUC, Balanced Accuracy, and any other reported metrics.

7. The manuscript would further benefit from reporting the average ranks obtained from the Friedman test. Average ranks provide valuable insights into the relative performance differences among competing methods and are widely used to support the interpretation of statistical comparison results. Including this information would strengthen the experimental analysis and improve the interpretability of the findings.

Reviewer #5: The author has validated the proposed results using both performance and statistical metrics. Since the necessary revisions have been incorporated into the manuscript, the paper is recommended for acceptance for publication.

Reviewer #6: The paper presents a genuine improvement and is suitable for publication. The theoretical derivation is now strong, the experimental section has been substantially expanded, and the statistical validation supports the claims.

**********

what does this mean?). If published, this will include your full peer review and any attached files.

If you choose “no”, your identity will remain anonymous but your review may still be made public.

**Do you want your identity to be public for this peer review?**  For information about this choice, including consent withdrawal, please see our Privacy Policy

Reviewer #1: No

Reviewer #2: **Yes:** Mohammad Khanbabaei

Reviewer #3: No

Reviewer #5: No

Reviewer #6: No

**********

[NOTE: If reviewer comments were submitted as an attachment file, they will be attached to this email and accessible via the submission site. Please log into your account, locate the manuscript record, and check for the action link "View Attachments". If this link does not appear, there are no attachment files.]

To ensure your figures meet our technical requirements, please review our figure guidelines: https://journals.plos.org/plosone/s/figures

You may also use PLOS’s free figure tool, NAAS, to help you prepare publication quality figures: https://journals.plos.org/plosone/s/figures#loc-tools-for-figure-preparation.

NAAS will assess whether your figures meet our technical requirements by comparing each figure against our figure specifications.

Attachment Submitted filename: comments_for_revised_manu.docx

# Author comment: Author response to Decision Letter 2

**DOI:** 10.1371/journal.pone.0356889.r004

21 Jul 2026

Response to Reviewers

Title: ASWBoost: A Robust Boosting Algorithm for Noisy and Imbalanced Classification via Parameterized Exponential Loss

Manuscript ID: PONE-D-25-60888R1

Journal: PLOS ONE

Article Type: Research Article

Corresponding Author: Mei Yan

First Author: Fei Meng

Order of Authors: Fei Meng; Mei Yan; Hang Liu; Wan Xu; Hao Li

Message to the Academic Editor

Dear Academic Editor, Dr. Kaywan Othman Ahmed,

We would like to extend our most sincere gratitude to you for handling our manuscript, "ASWBoost: A Robust Boosting Algorithm for Noisy and Imbalanced Classification via Parameterized Exponential Loss" (PONE-D-25-60888R1). We deeply appreciate the efficient, professional, and rigorous editorial work you have dedicated to this submission.

Thank you for coordinating such an excellent panel of reviewers and for granting us the valuable opportunity to revise our paper. Guided by your decision letter and the reviewers' constructive feedback, our entire research team has made our utmost effort to fundamentally upgrade the manuscript. We have conducted a comprehensive structural overhaul, significantly expanded the theoretical and experimental analyses, and rigorously enhanced the statistical validations to ensure the highest scientific quality.

We are truly grateful for your time, patience, and unwavering commitment to maintaining the high standards of the journal. We sincerely hope that our comprehensive revisions successfully address all concerns and that the refined manuscript is now deemed acceptable for publication in PLOS ONE. We wish you great health, joy, and continued success in all your endeavors!

Sincerely,

The Authors

Dear Academic Editor and Reviewers,

Thank you very much for your thorough evaluation of our manuscript and for providing such insightful and constructive feedback. We deeply appreciate the time and expertise you have dedicated to reviewing our work. Your comments have been invaluable in helping us significantly elevate the theoretical depth, structural clarity, and statistical rigor of our paper.

We have carefully considered each of the points raised and have extensively revised the manuscript accordingly. Below, we provide a detailed, point-by-point response to all comments from the Editor and the Reviewers. Corresponding modifications have been highlighted in the ‘Revised Manuscript with Track Changes’ document.

Best regards,

Mei Yan

Response to Reviewer #1

Point 1.1: Funding: The Financial Disclosure field states, “The author(s) received no specific funding for this work” while the Funding section identifies Grant No. 202405AS350003. These statements are contradictory and must be reconciled before publication.

Point taken.

Response:

We sincerely apologize for this administrative oversight during the initial online submission process. The information provided in the manuscript's “Funding” section is correct: this work was indeed supported by the Cross-integration Innovation Team of Modern Applied Mathematics and Life Sciences in Yunnan Province, China (Grant No. 202405AS350003).

To perfectly reconcile this contradiction and comply with PLOS ONE guidelines, we have taken the following corrective actions:

We have updated the “Financial Disclosure” field in the Editorial Manager system during this resubmission to accurately reflect the aforementioned grant.

We have explicitly included the corrected and updated Financial Disclosure statement in our Cover Letter.

Point 1.2: Abstract: The statement “ASWBoost outperforms standard AdaBoost and other mainstream ensemble algorithms” overstates the findings. Authors should consider naming the specific ensemble algorithms over which ASWBoost demonstrates statistically significant superiority and qualify the claim accordingly.

Done.

Response:

We completely agree with your assessment. Broad claims without specific context can inadvertently overstate the findings. We have revised the Abstract to explicitly name the exact algorithms that ASWBoost significantly outperforms based on our rigorous statistical tests, and we have objectively qualified its performance relative to XGBoost.

Revision in Abstract: “Critically, statistical analyses confirm that ASWBoost significantly outperforms AdaBoost, CS-AdaBoost, GBDT, SMOTEBoost, and RUSBoost, with overall performance statistically comparable to XGBoost.”

We would like to express our most sincere gratitude for your continuous support and your meticulous review of our manuscript. We deeply appreciate your kind well wishes ("Good luck!") and your precise final suggestions regarding the funding disclosure and the abstract. Your exceptionally keen eye for detail has helped us correct the administrative oversight and perfect the final version of this paper. Thank you once again for your invaluable time, patience, and expertise throughout the entire review process. We wish you all the best in your future research and endeavors, along with great health and success!

Response to Reviewer #2

Dear Reviewer #2,

We would like to express our deepest gratitude for your meticulous review and your exceptional patience in evaluating our manuscript. We sincerely apologize that our previous revision did not fully grasp the depth of your highly valuable suggestions. We have taken your comments to heart, and over the past weeks, our entire research team has made our utmost effort to completely overhaul the manuscript. We have rigorously studied the literature you recommended, fundamentally re-architected our presentation structure, and thoroughly upgraded our figures. We hope you will find that these comprehensive modifications, which are explicitly detailed below and highlighted in the ‘Revised Manuscript with Track Changes’, thoroughly address your concerns and meet the rigorous standards of PLOS ONE.

Point 2.1: This paper presents a novel method to improve the performance of AdaBoost in environments with noisy and imbalanced data. The paper claims novelty in improving AdaBoost; however, the authors should compare their proposed method with two similar studies listed below: 1. A noise-detection based AdaBoost algorithm for mislabeled data. 2. Bounded exponential loss function based AdaBoost ensemble of OCSVMs. If the proposed method is similar to those presented in the two above-mentioned articles, the authors should clearly explain the similarities and differences.

Response: Thank you for directing us to these two foundational and state-of-the-art studies. We completely agree that a rigorous mathematical and conceptual comparison

with these specific methods is essential to properly articulate the exact novelty of our ASWBoost algorithm. To thoroughly resolve this, we have incorporated your suggestions into two critical parts of our revised manuscript:

In the Introduction (Section 1): We have explicitly introduced these two studies to outline the current trajectory of robust boosting research. We acknowledged their significant contributions in mitigating AdaBoost's vulnerability to outliers and mislabeled data by preventing unbounded weight growth. This sets the stage for our motivation.

In the newly added theoretical section (Section 3.5: Connection and Distinction with Existing Robust Boosting Frameworks): We provided an in-depth, dedicated comparison detailing the exact similarities and differences:

Differences from ND-AdaBoost (Cao et al., 2012): ND-AdaBoost utilizes explicit noise-detection functions (e.g., via k-NN or EM algorithms) embedded into the loss function to assign noise labels iteratively. While highly effective, this explicit pre-filtering incurs substantial computational overhead. In contrast, ASWBoost achieves robust weight adjustment implicitly by parametrically reshaping the exponential loss via , avoiding the need for external distance metrics and maintaining higher algorithmic efficiency.

Differences from BELF-AEOCSVMs (Xing et al., 2024): Xing et al. elegantly introduced a bounded exponential loss function to cap the penalty for outliers. However, as mathematically demonstrated in their study, their modified loss function is non-convex, which necessitates complex numerical methods (like the Newton-Raphson approach) for optimization. ASWBoost, conversely, strictly preserves the desirable convexity of the exponential loss function, thereby guaranteeing stable global convergence for standard multi-class imbalanced classification tasks.

Point 2.2: In addition, the experimental results section should be improved. For example, the authors should describe the dataset in detail and explain the nature of the noise and class imbalance present in the data.

Response: We deeply appreciate this insightful suggestion. We fully agree that explicitly clarifying the nature of the data is crucial for readers to understand why ASWBoost is practically valuable. We have substantially expanded Section 4.4 (Performance on OpenML Datasets) to detail these intrinsic characteristics:

Nature of Class Imbalance: We explicitly explained that the severe class imbalance in the selected medical datasets (e.g., Mammography, Hypothyroid) stems from the natural epidemiological rarity of positive disease instances relative to healthy baselines.

Nature of Noise: We clarified that the noise in these real-world datasets is dual-faceted: it comprises feature noise (e.g., instrumental artifacts or sensor inaccuracies) and label noise (e.g., driven by subjective diagnostic discrepancies among practitioners).

We further linked these characteristics to our algorithm, explaining precisely how ASWBoost’s parametrically modified loss function dynamically caps the weight penalties on these overlapping, anomalous samples, preventing the model from memorizing noise.

Point 2.3: To further improve the clarity of the paper, the authors are encouraged to review the two above-mentioned articles and follow their presentation structure.

Response: Thank you for this highly professional guidance. We carefully reviewed the presentation paradigm of Cao et al. (2012) and Xing et al. (2024) and realized that our previous draft mixed background knowledge with theoretical derivations, which hindered clarity. To provide a top-tier reading experience, we have performed a massive structural overhaul of the entire manuscript:

Added a “Preliminaries” Section (Section 2): We isolated the standard AdaBoost algorithm (2.1) and the standard exponential loss definitions (2.2) to clearly separate existing knowledge from our novel contributions.

Consolidated Methodology and Theory (Section 3): We restructured the proposed ASWBoost into a highly logical flow: starting with the parametric loss function (3.1), deriving update formulae (3.2), proving the error bounds theoretically (3.3), explaining the algorithm's mechanism (3.4), and concluding with the aforementioned theoretical comparison to existing frameworks (3.5).

Refined Experimental Layout (Section 4): We reorganized the experiments into dedicated sub-sections (Synthetic data, Real-world data, Complexity, and Statistical Significance Analysis) to provide a systemic validation.

Point 2.4: The results should be presented using figures and charts to enhance clarity. Furthermore, the quality of the figures should be improved.

Response: We fully accept this criticism and have taken decisive actions to elevate the visual quality and clarity of our manuscript:

Transforming Tables to Charts: Following your advice to enhance visual clarity, we converted the previously tabular G-mean results into a highly intuitive bar chart (now Fig 6). This allows readers to directly and visually compare the performance trends across AUC, Balanced Accuracy, and G-mean simultaneously.

Upgrading Figure Quality to Publication Standards: We discarded all previous low-resolution plots. Our team utilized Python (matplotlib) to completely redraw every single figure (Figures 2, 3, 4, 5, 6, and 7). All figures have been exported in lossless TIFF format with ultra-high resolution (300 to 600 DPI), ensuring crisp fonts, standardized layouts, and absolute clarity, fully meeting the stringent publication requirements of PLOS ONE.

We cannot overstate how much your rigorous and constructive feedback has improved the caliber of our research. We have poured our utmost dedication into this revision to ensure every single one of your concerns has been thoroughly and scientifically resolved. We sincerely hope that you find this heavily revised manuscript satisfactory and acceptable for publication.

Response to Reviewer #3

Dear Reviewer #3,

We would like to express our deepest gratitude for your thorough, highly professional, and constructive review of our manuscript. We are particularly grateful for your encouraging remarks regarding the substantial improvements in our organization and methodology descriptions. Your meticulous attention to detail—ranging from figure labels and dataset characteristics to the rigorousness of our statistical analyses—has been instrumental in elevating our manuscript to a much higher scientific standard.

We have made our utmost effort to carefully address each of your insightful comments. Below, please find our detailed, point-by-point responses. The corresponding modifications have been highlighted in the ‘Revised Manuscript with Track Change’.

Point 3.1: In Figure 2, the label of the x-axis appears to be incorrect. Based on the context and the experimental setup, the x-axis should be related to the level of noise interference. The authors are encouraged to verify and correct the axis description.

Response: Thank you for catching this error with such careful observation. You are completely correct. The x-axis in Figure 2 was inadvertently mislabeled during the drafting process. We have verified and corrected the x-axis label in the revised Figure 2 to accurately read “Noise Level (%)” instead of the previous incorrect description.

Point 3.2: For Table 2, it would be beneficial to provide additional information regarding the datasets, including the number of classes and the number of instances in each class. Such information would help readers better understand the characteristics and imbalance level of the datasets used in the experiments.

Response: We sincerely appreciate this constructive suggestion. We fully agree that providing the exact instance distribution and the number of classes is crucial for readers to accurately comprehend the intrinsic complexity and the precise imbalance severity of the datasets used.

Accordingly, we have substantially expanded Table 2 (in Section 4.4) in the revised manuscript. We added new columns to explicitly report the “Original Classes”, “Majority Instances (Negative Class)”, and "Minority Instances (Positive

Class)". Furthermore, we updated the introductory paragraph in Section 4.4 to explain how multi-class datasets (e.g., Hypothyroid, Cardiotocography, and Dermatology) were converted into highly imbalanced binary classification tasks for this specific study, ensuring maximum clarity and transparency.

Point 3.3: The presentation of the experimental results is currently inconsistent across evaluation metrics. Specifically, G-mean scores are reported in tabular form (Table 3), whereas AUC and Balanced Accuracy are presented only through bar charts. To improve consistency and facilitate a more precise comparison among methods, it is recommended that all evaluation metrics be reported in tabular form. This would allow readers to clearly examine the exact performance values achieved by each method.

Response: We sincerely thank you for pointing out this inconsistency. We completely agree that a consistent presentation format across all evaluation metrics is absolutely essential for a high-quality paper, and we deeply respect your request to facilitate a precise numerical comparison among methods.

While we initially intended to convert all figures into tables as you recommended, another reviewer (Reviewer #2) explicitly requested the exact opposite: “The results should be presented using figures and charts to enhance clarity”.

To strike an optimal balance and perfectly reconcile both expert opinions, we have adopted the following comprehensive solution:

Consistent Graphical Presentation: We have standardized the format by converting the original G-mean table into a high-reso

Attachment Submitted filename: Response_to_Reviewers_auresp_2.docx

# Peer review: Decision Letter 2

**DOI:** 10.1371/journal.pone.0356889.r005

**Authors:** Kaywan Othman Ahmed

10 Aug 2026

ASWBoost: Classification Algorithm for Noisy and Imbalanced Data Based on Parametric Exponential Loss

PONE-D-25-60888R2

Dear Dr. Yan,

We’re pleased to inform you that your manuscript has been judged scientifically suitable for publication and will be formally accepted for publication once it meets all outstanding technical requirements.

Within one week, you’ll receive an e-mail detailing the required amendments. When these have been addressed, you’ll receive a formal acceptance letter and your manuscript will be scheduled for publication.

An invoice will be generated when your article is formally accepted. Please note, if your institution has a publishing partnership with PLOS and your article meets the relevant criteria, all or part of your publication costs will be covered. Please make sure your user information is up-to-date by logging into Editorial Manager at Editorial Manager® and clicking the ‘Update My Information' link at the top of the page. For questions related to billing, please contact billing support.

If your institution or institutions have a press office, please notify them about your upcoming paper to help maximize its impact. If they’ll be preparing press materials, please inform our press team as soon as possible -- no later than 48 hours after receiving the formal acceptance. Your manuscript will remain under strict press embargo until 2 pm Eastern Time on the date of publication. For more information, please contact onepress@plos.org.

Kind regards,

Kaywan Othman Ahmed

Academic Editor

PLOS One

Additional Editor Comments (optional):

Reviewers' comments:

Reviewer's Responses to Questions

**Comments to the Author**

Reviewer #2: All comments have been addressed

Reviewer #3: All comments have been addressed

**********

2. Is the manuscript technically sound, and do the data support the conclusions?

Reviewer #2: Yes

Reviewer #3: Yes

**********

3. Has the statistical analysis been performed appropriately and rigorously?

Reviewer #2: Yes

Reviewer #3: Yes

**********

4. Have the authors made all data underlying the findings in their manuscript fully available?

The PLOS Data policy

Reviewer #2: Yes

Reviewer #3: Yes

**********

5. Is the manuscript presented in an intelligible fashion and written in standard English?

Reviewer #2: Yes

Reviewer #3: Yes

**********

Reviewer #2: (No Response)

Reviewer #3: All of my comments have been addressed appropriately by the authors. Therefore, I recommend accepting the manuscript.

**********

what does this mean?). If published, this will include your full peer review and any attached files.

If you choose “no”, your identity will remain anonymous but your review may still be made public.

**Do you want your identity to be public for this peer review?**  For information about this choice, including consent withdrawal, please see our Privacy Policy

Reviewer #2: **Yes:** Mohammad Khanbabaei

Reviewer #3: No

**********

# Editor report: Acceptance letter

**DOI:** 10.1371/journal.pone.0356889.r006

**Authors:** Kaywan Othman Ahmed

PONE-D-25-60888R2

PLOS One

Dear Dr. Yan,

I'm pleased to inform you that your manuscript has been deemed suitable for publication in PLOS One. Congratulations! Your manuscript is now being handed over to our production team.

At this stage, our production department will prepare your paper for publication. This includes ensuring the following:

* All references, tables, and figures are properly cited

* All relevant supporting information is included in the manuscript submission,

* There are no issues that prevent the paper from being properly typeset

You will receive further instructions from the production team, including instructions on how to review your proof when it is ready. Please keep in mind that we are working through a large volume of accepted articles, so please give us a few days to review your paper and let you know the next and final steps.

Lastly, if your institution or institutions have a press office, please let them know about your upcoming paper now to help maximize its impact. If they'll be preparing press materials, please inform our press team within the next 48 hours. Your manuscript will remain under strict press embargo until 2 pm Eastern Time on the date of publication. For more information, please contact onepress@plos.org.

You will receive an invoice from PLOS for your publication fee after your manuscript has reached the completed accept phase. If you receive an email requesting payment before acceptance or for any other service, this may be a phishing scheme. Learn how to identify phishing emails and protect your accounts at https://explore.plos.org/phishing.

If we can help with anything else, please email us at customercare@plos.org.

Thank you for submitting your work to PLOS One and supporting open access.

Kind regards,

PLOS ONE Editorial Office Staff

on behalf of

Dr. Kaywan Othman Ahmed

Academic Editor

PLOS One
